# Rationally Designed Dual Kinase Inhibitors for Management of Obstructive Sleep Apnea—A Computational Study

**DOI:** 10.3390/biomedicines14010181

**Published:** 2026-01-14

**Authors:** Kosi Gramatikoff, Miroslav Stoykov, Mario Milkov

**Affiliations:** 1 Research Institute, Medical University “Prof. Dr. Paraskev Stoyanov”, 55 Marin Drinov Str., 9002 Varna, Bulgaria; 2 Department of Dental Material Science and Prosthetic Dental Medicine, Faculty of Dental Medicine, Medical University “Prof. Dr. Paraskev Stoyanov”, 84 Tsar Osvoboditel Blvd, 9002 Varna, Bulgaria; miroslav.stoikov93@gmail.com

**Keywords:** obstructive sleep apnea, comorbidities, rational drug design, dual kinase inhibitors, network pharmacology, casein kinase 1 delta (CK1δ), PINK1, *Nigella sativa*, plant alkaloids, neurodegeneration, precision therapeutics

## Abstract

**Background/Objectives:** Obstructive sleep apnea (OSA) affects approximately 1 billion adults worldwide with extensive comorbidities, including cardiovascular disease, metabolic disorders, and cognitive decline, yet pharmacological therapies remain limited. Conventional bottom-up omics approaches identify numerous genes overlapping with other diseases, hindering therapeutic translation. This study introduces a top-down, comorbidity-driven approach to identify actionable molecular targets and develop rational dual kinase inhibitors for OSA management. **Methods:** We implemented a five-tier modeling workflow: (1) comorbidity network analysis, (2) disease module identification through NetworkAnalyst, (3) mechanistic pathway reconstruction of the CK1δ-(HIF1A)-PINK1 signaling cascade, (4) molecular docking analysis of *Nigella sativa* alkaloids and reference inhibitors (IC261, PF-670462) against CK1δ (PDB: 3UYS) and PINK1 (PDB: 5OAT) using AutoDock Vina, and (5) rational design and computational validation of novel dual inhibitors (ICL, PFL) integrating pharmacophoric features from natural alkaloids and established kinase inhibitors. **Results:** Extensive network analysis revealed a discrete OSA disease module centered on two interconnected protein kinases—CK1δ and PINK1—that mechanistically bridge circadian disruption and neurodegeneration. Among natural alkaloids, Nigellidine showed strongest CK1δ binding (−8.0 kcal/mol) and Nigellicine strongest PINK1 binding (−8.6 kcal/mol). Rationally designed dual inhibitors demonstrated superior binding: ICL (−7.2 kcal/mol PINK1, −8.9 kcal/mol CK1δ) and PFL (−10.8 kcal/mol CK1δ, −11.2 kcal/mol PINK1), representing −2.6–2.8 kcal/mol improvements over reference compounds. **Conclusions**: This study establishes a comorbidity-driven translational framework identifying the CK1δ-PINK1 axis as a therapeutic target in OSA. The rationally designed dual inhibitors represent third-generation precision therapeutics addressing OSA’s multi-dimensional pathophysiology, while the five-tier workflow provides a generalizable template for drug discovery in complex multimorbid diseases.

## 1. Introduction

Obstructive sleep apnea [OSA] is a significant public health challenge affecting nearly 1 billion adults worldwide [1,2]. It contributes heavily to cardiovascular disease, metabolic disorders, and neurodegenerative conditions [1,2]. The complexity of OSA arises from an intricate web of comorbidities, including hypertension, stroke, and cognitive decline [3,4]. These interconnected pathologies often defy simple therapeutic intervention [3,4].

Network biology has revolutionized our understanding of these multimorbidities [5,6]. By revealing how distinct conditions share molecular mechanisms, it suggests that targeting shared pathways may yield superior clinical outcomes [5,6]. As Hu and colleagues demonstrated, diseases with shared genetic architectures often co-occur [5]. Mapping these “disease modules” allows for the identification of targets that address multiple pathological dimensions simultaneously [5].

Despite decades of research, a gap persists between molecular knowledge and effective pharmacological therapies. Current OSA management relies on mechanical interventions like CPAP, while drug options remain limited and symptomatic [7,8]. This translational deficit is partly due to traditional “bottom-up” approaches [9,10]. These omics-scale methods often identify hundreds of genes that overlap with other diseases, making it difficult to find targets specific to OSA [9,10]. While systems biology has shown promise, its application to OSA is hindered by the lack of well-defined molecular signatures [11,12].

### 1.1. A Top-Down, Comorbidity-Driven Strategy

This study introduces a fundamentally different strategy: a top-down, comorbidity-driven approach. This investigation has four major implications for precision medicine.

First, we establish a novel data integration framework [13,14]. By beginning with clinically validated comorbidities and working backward, we prioritize targets with established relevance over non-specific omics findings [13,14]. This directly addresses the challenges of disease module identification in multimorbid conditions [15,16].

Second, our analysis identifies a previously unrecognized disease module centered on two kinases: casein kinase 1 delta [CK1δ] and PTEN-induced kinase 1 [PINK1] [17,18]. These kinases bridge two critical OSA domains: circadian rhythm disruption and neurodegeneration [17,18]. While the link between OSA and cognitive decline is known, the molecular connection has remained elusive [19,20]. We demonstrate that CK1δ [a circadian regulator] and PINK1 [a mitochondrial quality control kinase] are interconnected through shared regulators and the HIF1A signaling pathway [21,22,23,24].

Third, we evaluate natural compounds from *Nigella sativa* [black seed] [25,26]. While crude extracts show efficacy in OSA, their individual active constituents remain uncharacterized [25,26]. We use computational chemistry to identify specific alkaloids responsible for dual CK1δ/PINK1 modulation. We then advance to design first-generation, rationally optimized dual inhibitors, ICL and PFL [27,28]. These compounds integrate features from established inhibitors like IC261 and PF-670462 to achieve superior binding affinities [27,28].

**Figure 1:**
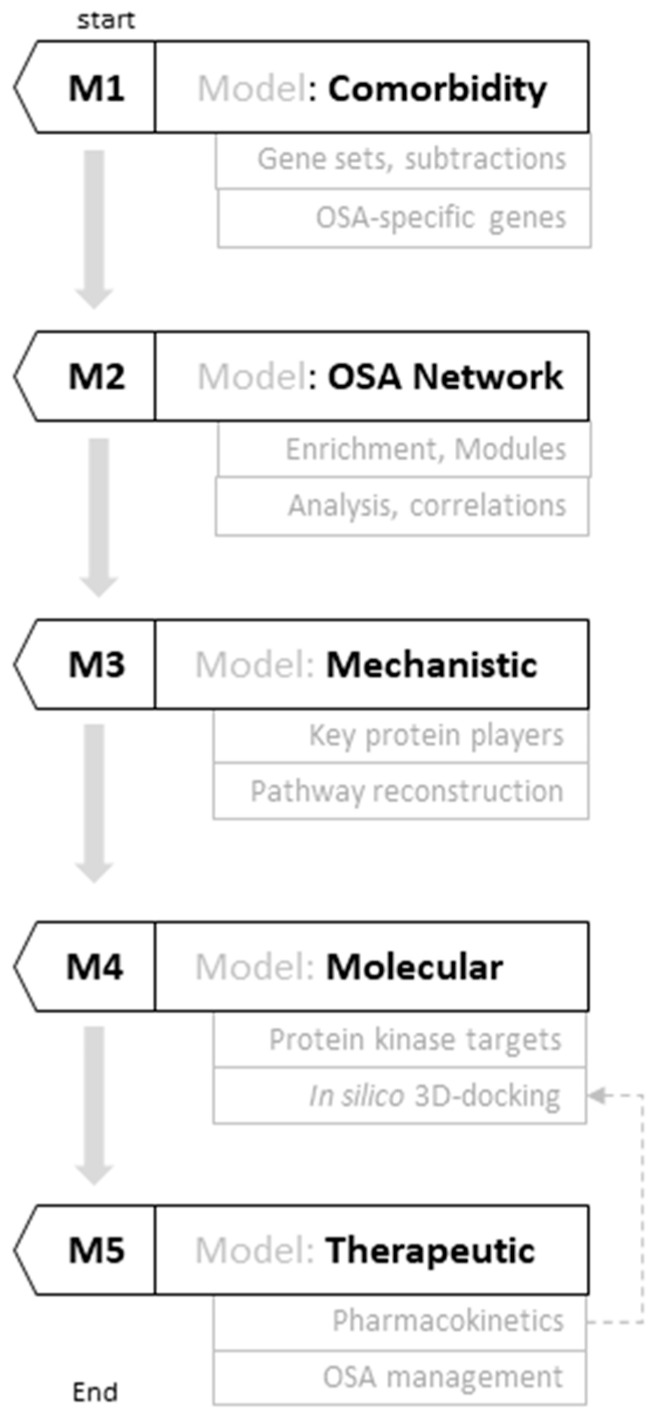
Overview of the five-tier workflow of models used in this study. The workflow begins with the collection and integration of disease-related genes, pathways, and molecular interactions from curated biomedical databases relevant to obstructive sleep apnea (OSA) (Step M1). These data were analyzed using network pharmacology approaches to construct interaction maps and identify central kinases and convergent signaling axes implicated in OSA pathophysiology (Step M2). Insights from this systems-level analysis guided the rational design of dual-kinase inhibitor hybrids by combining pharmacophoric elements from selected natural alkaloids and reference drug scaffolds (Step M3). The resulting hybrid structures were evaluated through molecular docking and structural modeling to characterize predicted binding interactions with both target kinases and to prioritize lead candidates (Step M4). Step M5 integrates comprehensive pharmacokinetic profiling, including in silico ADME (Absorption, Distribution, Metabolism, and Excretion) prediction, drug-likeness assessment, toxicity screening, and evaluation of potential off-target liabilities to identify compounds with suboptimal pharmaceutical properties. Compounds exhibiting ADME deficiencies undergo iterative structure-guided optimization (feedback loop M5 → M4), wherein strategic chemical modifications address identified liabilities while preserving dual-kinase binding affinity, followed by re-docking validation to confirm retained target engagement (marked by a reverse arrow from M5 to M4). This iterative refinement cycle yields second-generation inhibitors with superior pharmacokinetic profiles, which are then integrated into a mechanistic therapeutic model proposing how coordinated dual-kinase modulation with pharmaceutically optimized agents can be leveraged for the pharmacological management of OSA.

Fourth, we present a five-tier modeling workflow to translate clinical data into a management model (Figure 1) [29,30]:

1. Comorbidity network analysis (Figure 2 and Figure 3).
2. Disease module identification (Figure 4 and Figure 5B).
3. Mechanistic pathway reconstruction (Figure 6A).
4. Natural product screening and rational drug design (Figure 7, Figure 8 and Figure 9).
5. Systems-level integration (Figure 10).

**Figure 2:**
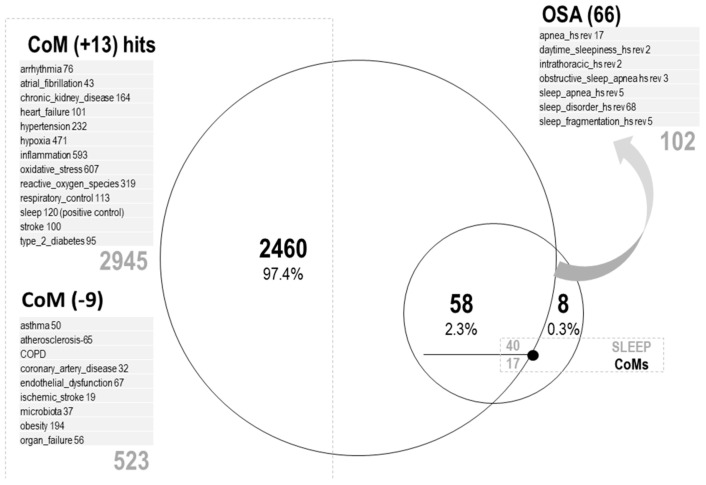
Collection and integration of disease-related genes (Step M1). This figure illustrates the initial data-integration stage in which gene sets associated with multiple OSA-related comorbidities (CoM) were compiled and compared against the pool of genes linked to obstructive sleep apnea (OSA). A Venn diagram summarizes the overlap and distinction between these datasets, with the non-overlapping segment representing a subset of 66 genes unique to the comorbidity groups after subtraction of genes already implicated in OSA. These 66 comorbidity-specific genes constitute the refined gene set used for subsequent network pharmacology analyses and downstream prioritization steps.

**Figure 3:**
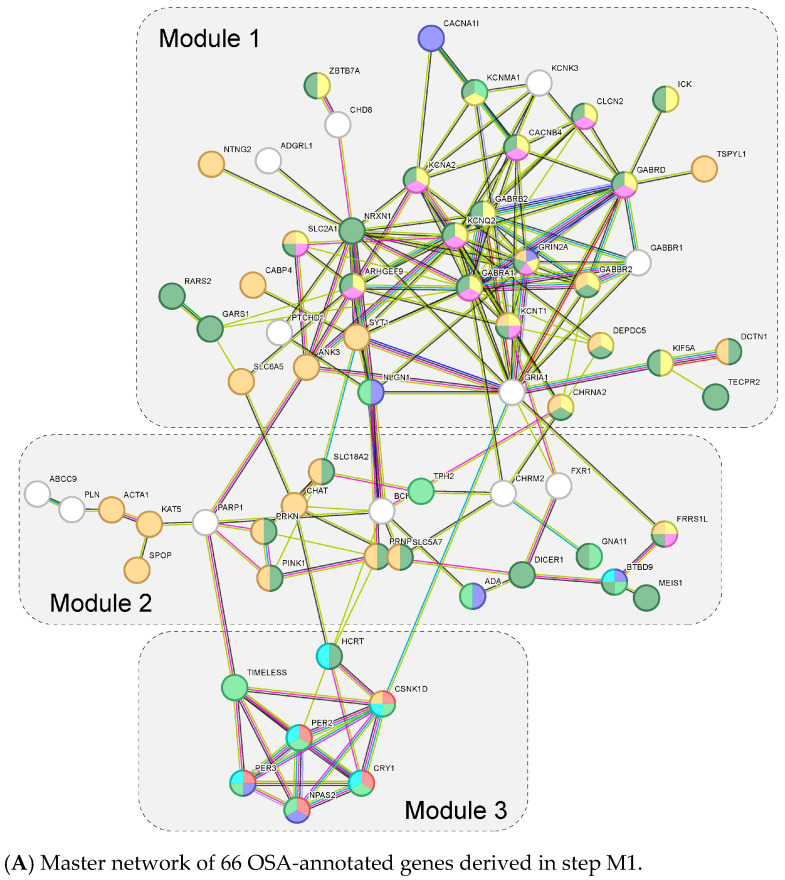

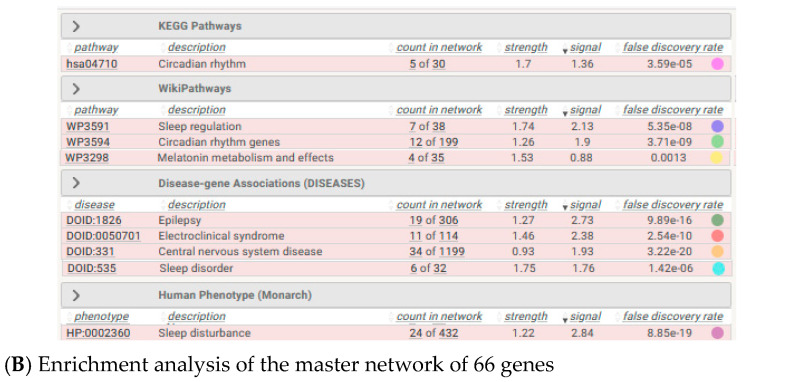
Master network and functional enrichment of 66 OSA-annotated genes derived in Step M1. (**A**) The network contains 171 edges, with an average node degree of 5.18 and an average local clustering coefficient of 0.505. It is composed of three sub-modules: Module 1, enriched in ion channels and containing two GPCRs; Module 2, designated the D-module for ‘dementia’ due to its inclusion of PINK1 and PRKN, known Parkinson’s-related genes, and acting as an interface to Module 3; and Module 3, labeled the C-module for its enrichment in circadian genes. The network was generated in STRING [31]. (**B**) Enrichment analysis showing a list of statistically significant ontologies selected to color the nodes in the master network. Module 1 shows enrichment in ion channel–related functions, including the GPCRs. Module 2 (D-module) highlights dementia- and Parkinson’s-related processes through PINK1 and PRKN (Serine/threonine protein kinase PINK1, UniProt ID: Q9BXM7). Module 3 (C-module) is enriched for circadian rhythm–related genes (controlled by Casein kinase I isoform delta CSNK1D/CK1 delta, UniProt ID: P48730), with the D-module acting as an interface between Modules 1 and 3, suggesting functional connectivity between ion channel regulation, neurodegeneration, and circadian control. Nodes are color-coded according to ontology categories: circadian genes are highlighted in bright green and pink, emphasizing their central role; the broader circadian rhythm ontology is marked in bright green to show interconnected processes; sleep regulation pathways are indicated in blue-purple to distinguish regulatory mechanisms; and disorder-related nodes are colored in cyan. The figure illustrates how key circadian genes integrate into circadian rhythm pathways, which in turn influence sleep regulation, while certain nodes link to associated disorders, reflecting the multilevel relationships between gene function, physiological processes, and disease phenotypes.

**Figure 4:**
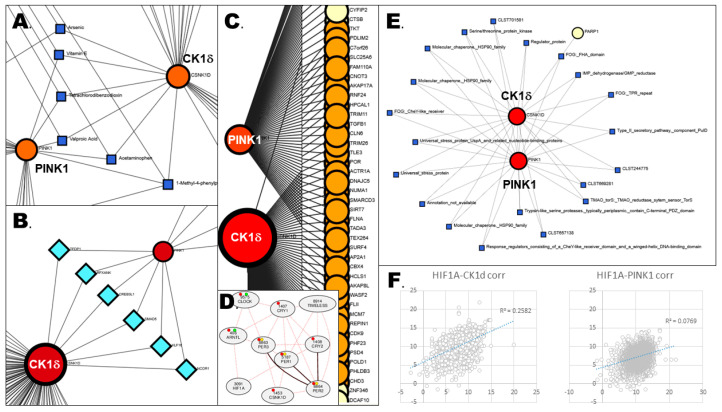
Network analysis reveals functional connections between CK1δ and PINK1 through shared compounds, transcription factors, and gene co-expression patterns. (**A**) Chemical compound-protein interaction network showing common natural and synthetic compounds that interact with both CK1δ (CSNK1D) and PINK1. Blue squares represent compounds including arsenic, vitamin C, phytoestrogen/genistein, acetaminophen, valproic acid, and 1-methyl-4-phenyl+. (**B**) Transcription factor regulatory network identifying shared transcriptional regulators of CK1δ and PINK1. Cyan diamonds represent transcription factors, including E2F1, FOXM1, CCNEB2-1, SMAD4, and YY1, that coordinately regulate both kinases. (**C**) Gene co-expression network in Jurkat cells demonstrating genes co-expressed with both PINK1 and CK1δ. The vertical gray-to-white gradient represents the spectrum of shared co-expressed genes (orange circles), with PINK1 and CK1δ (red circles) positioned on opposite sides. Key shared targets include CYPIP2, CTSB, APBB2, POLUM2, C7orf28, SLC25A8, FAM110A, CNOT3, NKAP17A, RNF24, IFPCAL1, TRIM11, TCFB1, CLN9, TRIM28, TLE3, PDOR, NCTR1A, DNAJC5, PRIMA1, SMARCD3, SIRT7, FLNA, TADA3, TEX264, SURF4, AP2A1, 2BX4, HCLS1, OSTRL, WASF2, FLII, CNP, RFRPN1, RABIF, PHF23, PSD4, POLD1, PHLDB3, CHD3, ZNF346, and DCAF10. (**D**) HIF1A-centered co-expression network derived from COXPRESSdb showing direct interactions between HIF1A and key metabolic regulators (ARNTL, BHLHE40/41, PER1/2, PER3, NPAS2, NR1D1, NR1D2, CSNK1D) involved in circadian and hypoxic responses. Red arrows indicate validated interactions, with dashed pink lines showing predicted associations. (**E**) Protein–protein interaction network highlighting functional proximity of CK1δ and PINK1 through shared protein domains, pathways, and microbiome-related interactions. Blue squares represent functional annotation clusters including CLST701561, Serine/threonine_protein_kinase, Regulator_protein, Molecular_chaperone_HSP90_family, FOG_FH2_domain, MAP_dehydrogenase/GMP_reductase, FOG_TPR_repeat, Type_II_secretory_pathway_component_PulD, Universal_stress_protein_UspA_and_related_nucleotide-binding_proteins, CLST244775, CLST086281, TMAO_lvrS-_TMAO_reductase_sytem_sensor_TorS, Trypsin-like_serine_proteases_typically_periplasmic_contain_C-terminal_PDZ_domain, CLST057138, Molecular_chaperone_HSP90_family, and Response_regulators_consisting_of_a_CheY-like_receiver_domain_and_a_winged-helix_DNA-binding_domain. PARP1 (white circle) serves as a bridging node. (**F**) Scatter plots showing positive correlations between HIF1A expression and both CK1δ ((**left**), R^2^ = 0.2582) and PINK1 ((**right**), R^2^ = 0.0769) based on co-expression data from COXPRESSdb, supporting transcriptional coordination between hypoxia signaling and the circadian/mitochondrial quality control axes.

**Figure 5:**
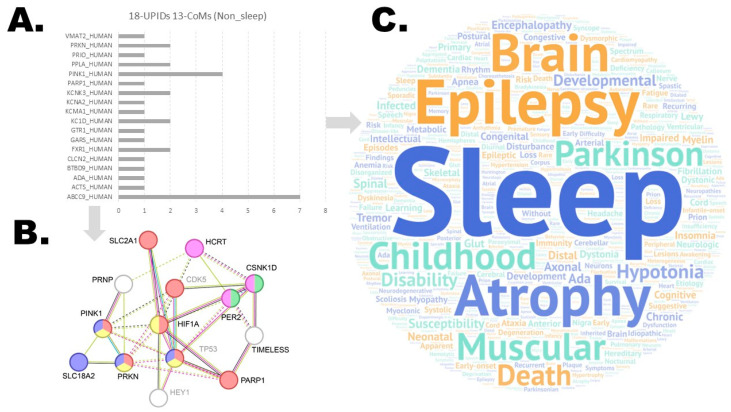
Identity of proteins in Module 2, their interconnection, and Mendelian word-cloud comorbidity analysis. (**A**) Bar plot representing the frequency with which each of the 18 proteins/genes (UniProts) identified in Step M2 appears across the 7 comorbidities (CoMs) identified in the upstream disease–gene mapping analysis. The height of each bar reflects the number of independent CoMs associated with the corresponding gene; for example, PINK1 appears in four distinct comorbidity categories, underscoring its recurrent involvement across disease contexts. Each gene is color-coded according to its dominant functional annotation: kinase-related or kinase-binding genes (red), mitophagy-associated genes (yellow), Parkinson’s disease–linked genes (blue), circadian rhythm regulators (green), and sleep-disorder-associated genes (pink) (**B**). Enriched hybrid “Module 2/3” derived from extended STRING network analysis, which revealed three additional high-centrality genes—HIF1A, TP53, and CDK5—not present in the original list but appearing as critical linking nodes between multiple functional clusters. These genes form isolated but topologically significant connectors that bridge mitochondrial stress pathways, cell-cycle or damage responses, and neurophysiological signaling layers. The integration of these three regulators into Module 2 highlights the convergence of kinase signaling, hypoxic transcriptional control, and neuronal resilience mechanisms. (**C**) Word-frequency cloud generated from the combined text of 26 OMIM entries associated with the 18 proteins/genes. A total of 154 unique high-frequency terms were extracted, with WordCloud-based weighting reflecting semantic prominence of disease-related major concepts such as “sleep,” “brain,” “Parkinson,” “neurodegeneration,” “mitochondria,” “hypoxia,” “dystonia,” “channelopathy,” and “autosomal,” capturing the shared pathological vocabulary across the phenotypes linked to the module. Together, these three panels summarize the development of Module 2 through comorbidity recurrence, network-based gene expansion, and phenotype-level text mining.

**Figure 6:**
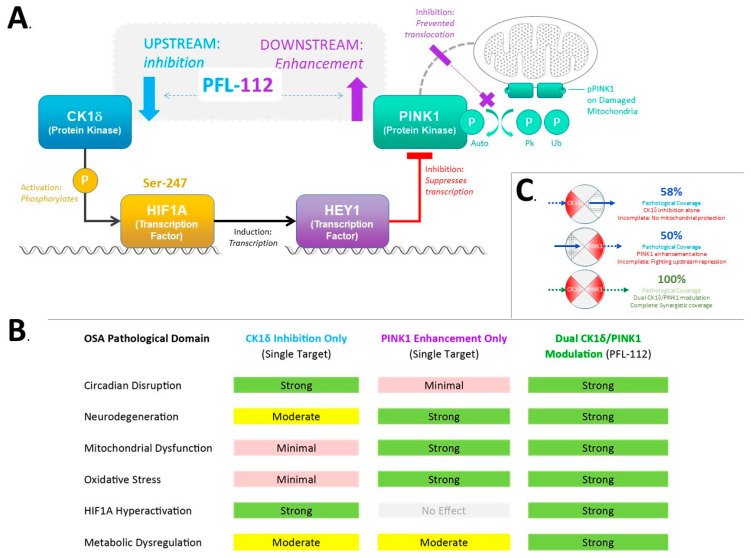
Reconstructed signaling cascade connecting CK1δ and PINK1 through HIF1A and HEY1 (Step M3). The pathway begins with CK1δ (blue box), a serine/threonine kinase that phosphorylates and activates HIF1A (orange box), consistent with published mechanistic data on CK1δ–HIF1A crosstalk. Activated, phosphorylated HIF1A (marked by an orange “P” circle) functions as an upstream transcription factor that induces HEY1 (purple box), a basic helix–loop–helix transcriptional repressor of the Hairy/Enhancer-of-split family. As reported previously [32], HEY1 directly represses PINK1 expression, thereby inhibiting the abundance of PINK1 required for mitochondrial import. PINK1 (teal box), a mitochondrial serine/threonine kinase essential for mitophagy and mitochondrial quality control, is shown adjacent to a dashed mitochondrial boundary, indicating the organelle compartment to which PINK1 normally translocates under stress conditions. The inhibitory effect of HEY1 on PINK1 prevents this mitochondrial translocation step, depicted as a gray dashed arrow crossed with a red bar. The diagram uses solid black arrows to denote activation or positive regulation (e.g., CK1δ → HIF1A, HIF1A → HEY1), red blunt-ended lines to indicate transcriptional repression (HEY1 ⊣ PINK1), and gray dashed arrows with red inhibitory bars to represent blocked cellular translocation events. Colored rectangular nodes with gradient fills distinguish each protein class, and a legend beneath the schematic provides a visual key to arrow styles, phosphorylation markings, and regulatory relationships. Importantly, this reconstructed axis represents a stress-responsive rather than constitutive signaling cascade. Under baseline physiological conditions, these nodes operate largely independently. However, in the context of OSA-associated chronic intermittent hypoxia, HIF1A stabilization coupled with CK1δ-mediated amplification creates an aberrant functional coupling that links circadian dysregulation with suppressed mitochondrial quality control through HEY1-mediated PINK1 repression. This stress-induced pathway integration provides mechanistic rationale for dual-kinase modulation as a strategy to disrupt maladaptive signaling convergence in OSA. Together, the pathway reconstruction highlights a mechanistic axis linking circadian kinase CK1δ with mitochondrial stress-response kinase PINK1 through oxygen-sensing and transcriptional repression layers, providing a unified explanatory model for kinase-mediated regulation relevant to OSA-associated cellular stress biology. (**A**): Dual-kinase “pincer strategy” demonstrating mechanistic complementarity. Schematic representation of the stress-inducible CK1δ → HIF1A → HEY1 ⊣ PINK1 signaling axis activated by chronic intermittent hypoxia characteristic of OSA pathophysiology. The cascade initiates with hypoxia-driven HIF1A stabilization, which is further amplified by CK1δ-mediated phosphorylation, leading to transcriptional induction of HEY1. HEY1 functions as a transcriptional repressor of PINK1, thereby suppressing mitochondrial quality control precisely when oxidative stress from hypoxia–reoxygenation cycles demands enhanced mitophagy. Dual CK1δ/PINK1 modulation, exemplified by the third-generation compound PFL-112, intervenes at two mechanistically complementary nodes, creating a “pincer strategy”: (1) Upstream intervention (blue): CK1δ inhibition prevents pathological HIF1A amplification, reducing downstream HEY1-mediated PINK1 repression while simultaneously realigning circadian rhythms and dampening neuroinflammation (temporal profile: preventive/modulatory, addressing the cause of dysregulation); (2) Downstream compensation (purple): PINK1 functional enhancement/preservation maintains mitochondrial quality control despite residual upstream stress, removing damaged mitochondria, reducing oxidative stress accumulation, and protecting neurons from cumulative hypoxic damage (temporal profile: protective/restorative, addressing the consequences of chronic damage). Blue and purple boxes detail specific mechanisms for each intervention point with checkmarks indicating validated effects. The key insight box (bottom) explains how this dual intervention creates a self-reinforcing beneficial cycle: upstream CK1δ inhibition relieves transcriptional suppression of PINK1, thereby enhancing the effectiveness of downstream mitochondrial protection. This complementary dual action addresses both the proximate cause (dysregulated signaling) and the ultimate consequence (mitochondrial damage) of chronic intermittent hypoxia in OSA. (**B**,**C**): Quantitative comparison of therapeutic coverage across pathological domains. Heat map visualization comparing the extent to which single-target versus dual-target therapeutic strategies address six major pathological domains characteristic of OSA: circadian disruption, neurodegeneration, mitochondrial dysfunction, oxidative stress, HIF1A hyperactivation, and metabolic dysregulation. Coverage scoring: gray = no effect; red = minimal effect; yellow = moderate effect; green = strong effect. Each domain is represented with a corresponding icon for visual clarity. ((Left column, blue header) CK1δ inhibition alone achieves strong effects on circadian disruption and HIF1A hyperactivation and moderate effects on neurodegeneration and metabolic regulation but provides minimal coverage of mitochondrial dysfunction and oxidative stress (aggregate coverage: 58%). ((Middle column, purple header) PINK1 enhancement alone achieves strong effects on neurodegeneration, mitochondrial dysfunction, and oxidative stress, with moderate effects on metabolic regulation, but provides no effect on HIF1A hyperactivation and minimal effect on circadian disruption (aggregate coverage: 50%). ((Right column, green header) Dual CK1δ/PINK1 modulation achieves strong effects across all six pathological domains (aggregate coverage: 100%), demonstrating comprehensive therapeutic potential. Summary statistics cards below the table quantify coverage percentages with visual indicators: red X marks denote incomplete coverage for single-target approaches (“No mitochondrial protection” for CK1δ-only; “Fighting upstream repression” for PINK1-only), while a green checkmark denotes complete synergistic coverage for dual targeting. The clinical implications box (bottom) contrasts single-target limitations (residual symptoms, incomplete protection, unaddressed domains) with dual-target advantages (comprehensive coverage, synergistic effects, self-reinforcing therapeutic cycle). This quantitative analysis demonstrates that polypharmacology targeting mechanistically complementary nodes provides superior therapeutic coverage for complex multisystem diseases like OSA compared to traditional single-target approaches. Abbreviations: CK1δ, casein kinase 1 delta; HIF1A, hypoxia-inducible factor 1-alpha; HEY1, hes-related family bHLH transcription factor with YRPW motif 1; PINK1, PTEN-induced kinase 1; OSA, obstructive sleep apnea.

**Figure 7:**
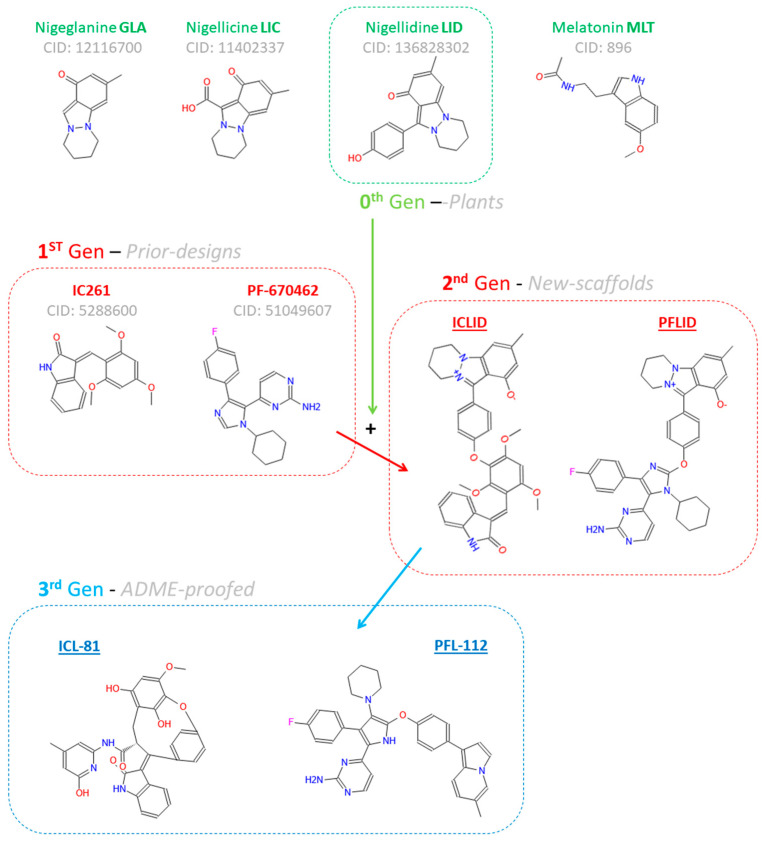
Multi-generational development and ADME-guided optimization of dual CK1δ/PINK1 inhibitors from natural product scaffolds to pharmaceutically optimized lead compounds. The figure illustrates a four-generation drug development workflow progressing vertically from natural alkaloid starting points (top, green) to ADME-validated therapeutic candidates (bottom, blue). The 0th generation (green, “Plants”) comprises natural alkaloids from *Nigella sativa* seeds serving as initial pharmacophoric templates: Nigeglanine (GLA, CID: 12116700), a piperidine-substituted pyridone; Nigellicine (LIC, CID: 11402337), an indazole alkaloid with carboxylic acid functionality; Nigellidine (LID, CID: 136828302, boxed), a complex tetracyclic indazoloquinoline selected as the primary natural scaffold; and Melatonin (MLT, CID: 896), included as a circadian-modulating reference compound. The 1st generation (red, “Prior-designs”) consists of established kinase inhibitors used as synthetic templates: IC261 (CID: 5288600), a benzofuran-based selective CK1δ inhibitor, and PF-670462 (CID: 51049607), a pyrazolopyrimidine dual CK1δ/CK1ε inhibitor. Green and red arrows converge to indicate rational combination of 0th and 1st generation pharmacophoric elements, yielding the 2nd generation (red, “New-scaffolds”): ICLID and PFLID, hybrid inhibitors designed for dual CK1δ/PINK1 targeting that demonstrated improved docking profiles but exhibited significant ADME liabilities, including poor solubility (Log S < −7), excessive lipophilicity (Log P > 5), low bioavailability scores (0.17–0.55), and structural alerts (quaternary nitrogens, Michael acceptors). A blue arrow indicates ADME-guided optimization whereby systematic structure modifications addressed these pharmaceutical deficiencies through strategic functional group replacements, molecular weight reduction, charge neutralization, and elimination of reactive moieties, followed by re-docking validation to confirm retained target binding affinity. This iterative refinement process produced the 3rd generation (blue, “ADME-proofed”): ICL-81 and PFL-112, third-generation leads exhibiting superior drug-likeness (Lipinski-compliant, bioavailability scores > 0.50), enhanced aqueous solubility (Log S > −6), elimination of structural alerts, and preserved dual-kinase inhibitory potential, thereby establishing pharmaceutically viable candidates for preclinical development in obstructive sleep apnea therapy. PubChem compound identifiers (CID) are provided for all reference compounds.

**Figure 8:**
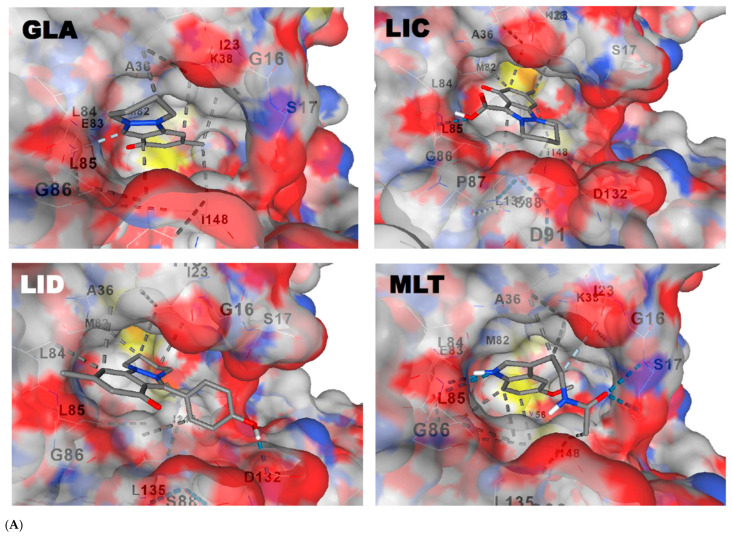

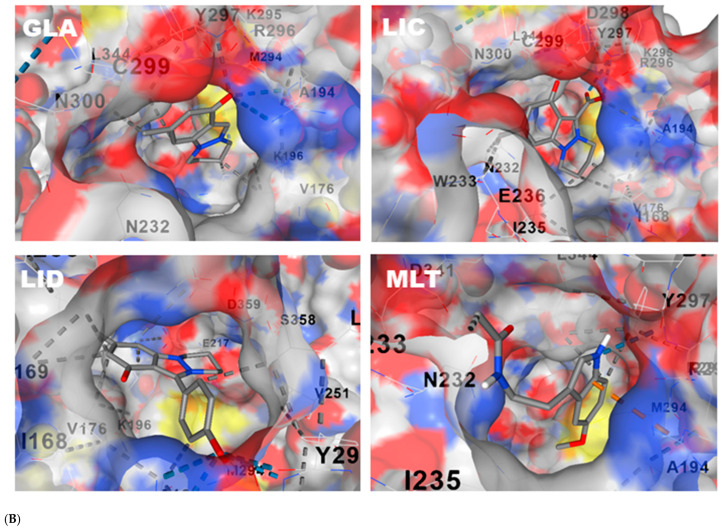
Molecular docking of Nigella sativa alkaloids and melatonin into CK1δ and PINK1 binding sites. Predicted binding modes of natural compounds in the ATP-binding pockets of (**A**) CK1δ (PDB: 3UYS) and (**B**) PINK1 (PDB: 5OAT). Compounds shown include Nigeglanine (GLA), Nigellicine (LIC), Nigellidine (LID), and Melatonin (MLT). Protein surfaces are colored according to electrostatic potential (red = negative, blue = positive, white = neutral). Key binding site residues are labeled. The compounds adopt distinct conformations within each binding pocket, with Nigellidine (LID) showing the most extensive interactions with both kinases. Dashed lines represent hydrogen bonds and other non-covalent interactions. The larger binding cavity of PINK1 compared to CK1δ accommodates different binding orientations of the alkaloid scaffolds [33,34,35].

**Figure 9:**
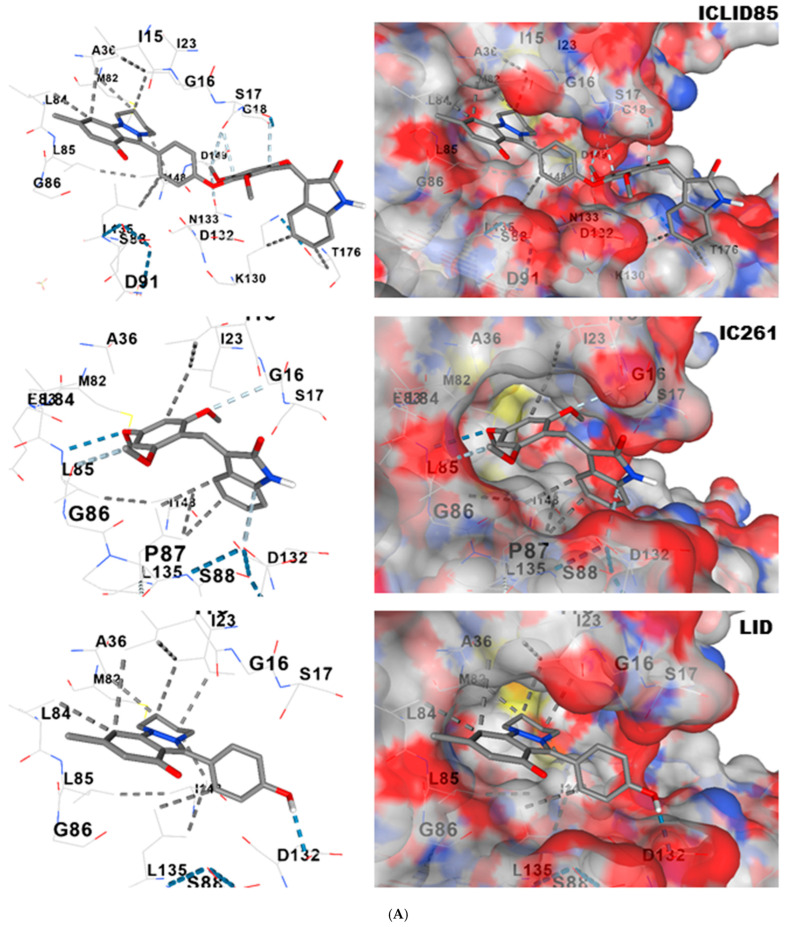

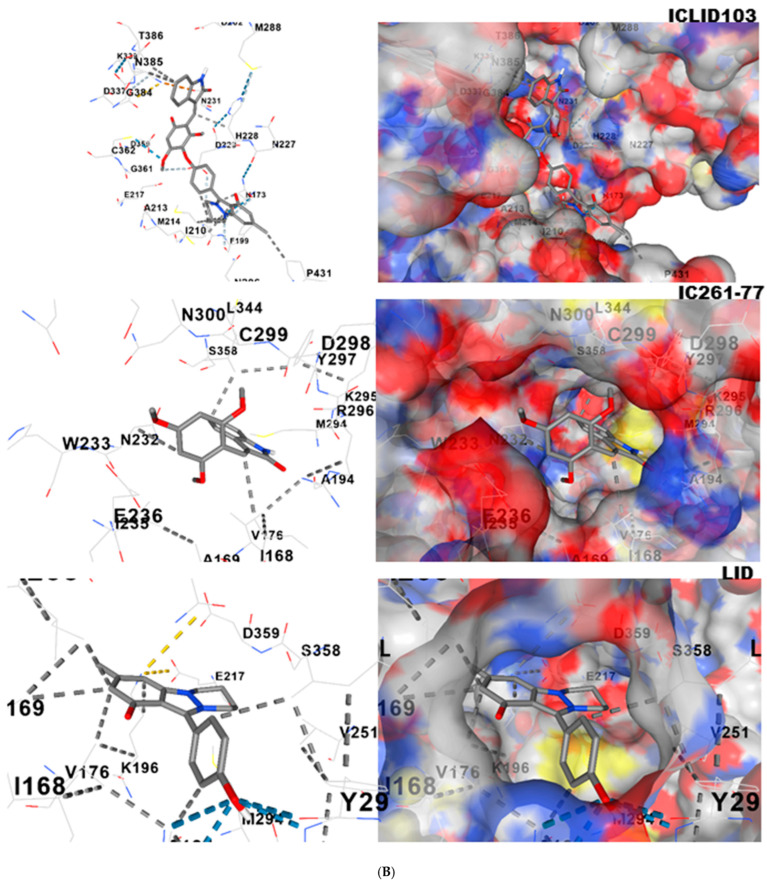

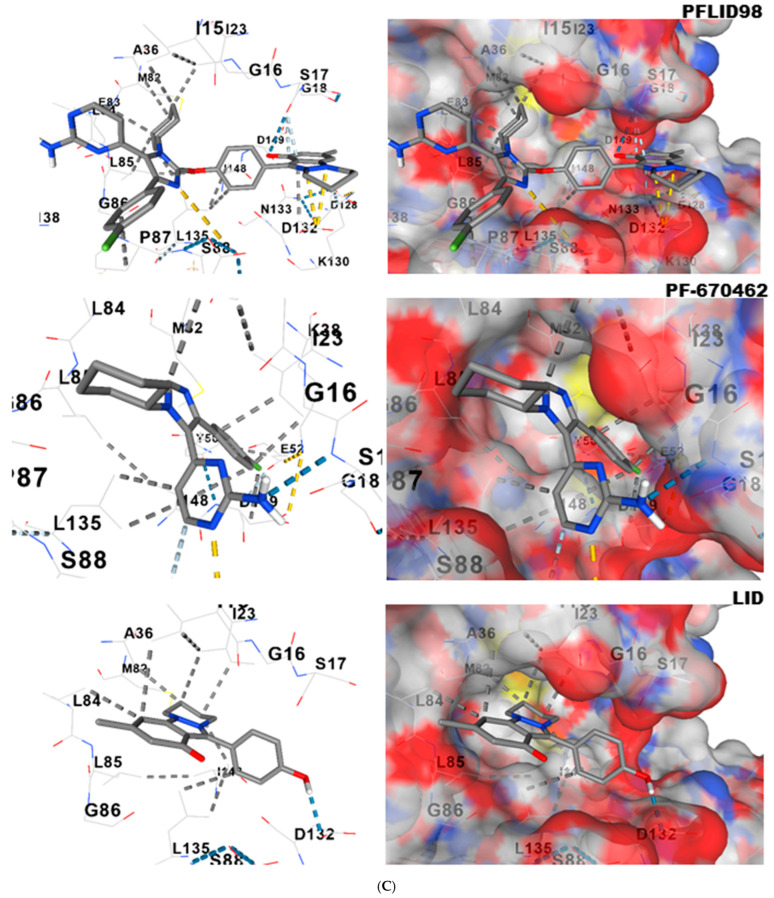

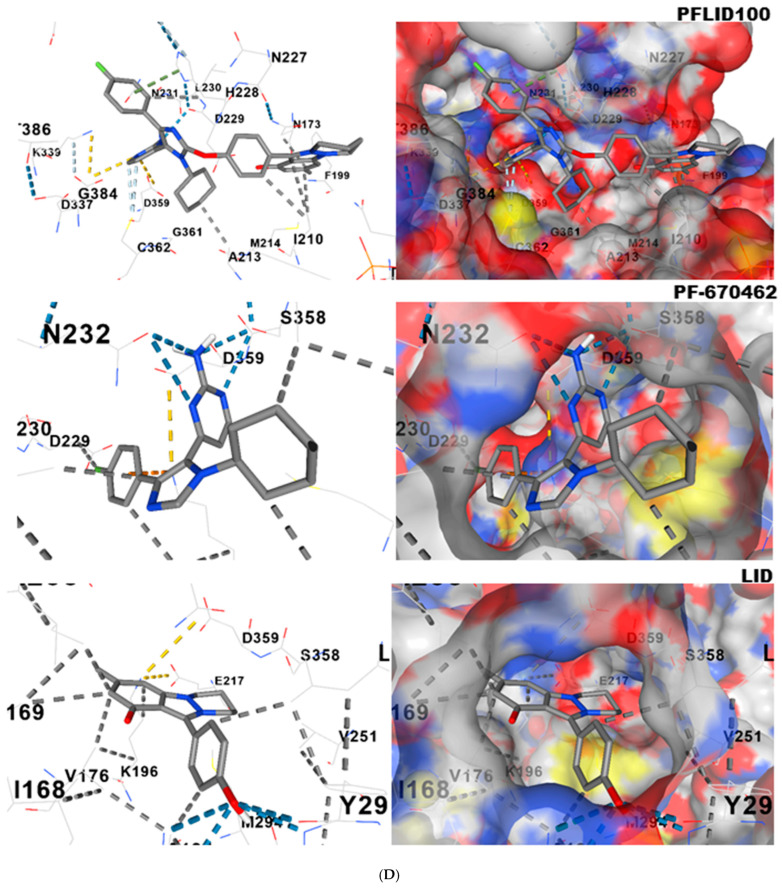

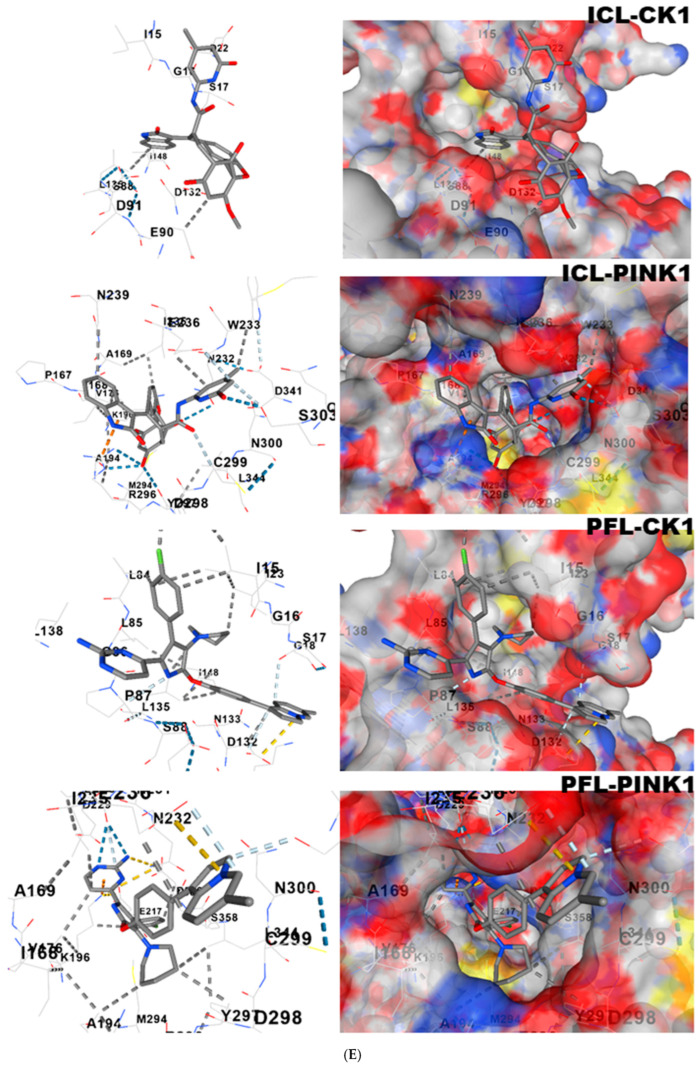
Comparative molecular docking of rationally designed dual inhibitors with reference compounds and natural alkaloids across multi-generational development. Binding mode analysis showing both stick representation with interaction networks (**left panels**) and electrostatic surface representation (**right panels**) for: (**A**) ICLID (same as ICLID85) and IC261 compared to Nigellidine (LID) in CK1δ; (**B**) ICLID103 (same as ICLID103) and IC261-77 compared to LID in PINK1; (**C**) PFLID (same as PFLID98) and PF-670462 compared to LID in CK1δ; (**D**) PFLID (same as PFLID100) and PF-670462 compared to LID in PINK1; and (**E**) third-generation ADME-optimized inhibitors ICL-81 and PFL-112 in both CK1δ and PINK1. The numerical suffixes (85, 103, 98, 100) indicate Vina binding scores in kcal/mol × 10. Left panels display detailed intermolecular interactions, including hydrogen bonds (blue dashed lines), hydrophobic contacts (gray dashed lines), and π-interactions with key binding site residues labeled. Right panels show the ligands (stick representation) positioned within the binding pocket surface, colored by electrostatic potential (red = negative, blue = positive, white = neutral). The rationally designed inhibitors (ICLID and PFLID variants) demonstrate optimized binding configurations that integrate favorable interactions from both parent reference inhibitors (IC261 and PF-670462) and natural alkaloid scaffolds. Key structural features of the ATP-binding pockets are highlighted, including the hinge region (CK1δ: L85, G86; PINK1: N232, D359), catalytic residues (CK1δ: D132, K38; PINK1: D359, K219), and hydrophobic pockets (CK1δ: I15, I23, L84, M82; PINK1: I168, I169, V176, Y291). The enhanced binding affinities of designed compounds result from improved shape complementarity, extended interaction networks with peripheral binding regions, and optimized hydrogen bonding patterns compared to reference inhibitors. (**E**) demonstrates that structure-guided ADME optimization preserved critical binding interactions while achieving superior pharmaceutical properties: ICL-81 maintains key contacts with CK1δ hinge residues (G16, S17) and the hydrophobic spine (I15, L84, L85) and engages PINK1 catalytic residues (D341, S309, N300) with extensive interactions involving the DFG motif region (D298, Y299) and hydrophobic contacts with A169, A194, and M294; PFL-112 forms an extensive hydrogen bonding network with CK1δ residues including S17, L85, D132, and N133 while occupying the hydrophobic cavity defined by I15, L84, P87, and I148, and establishes strong interactions with the PINK1 hinge region (E217, N239) and ATP-binding pocket (S309, S358, N300) with additional contacts to hydrophobic residues Y169, A169, M294, and the critical gatekeeper region near D298 and C299. Notably, PFL-112 achieved the highest predicted binding affinities for both targets (CK1δ: −10.8 kcal/mol; PINK1: −11.2 kcal/mol) while simultaneously exhibiting Lipinski-compliant properties, demonstrating that systematic ADME-guided optimization can enhance rather than compromise target engagement. Yellow spheres in surface representations indicate favorable hydrophobic contact regions.

**Figure 10:**
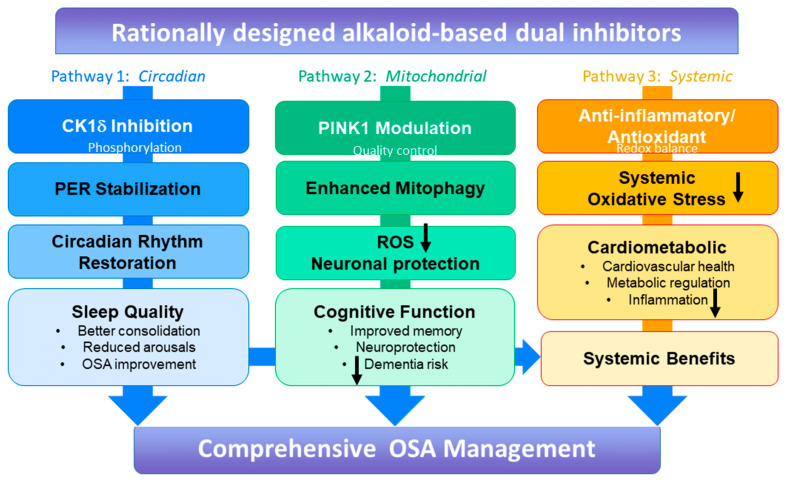
Mechanistic model for alkaloid-based OSA treatment. This diagram illustrates a multi-target therapeutic approach utilizing rationally designed dual inhibitors based on *Nigella* alkaloids (ICLID, PFLID and others) for the management of obstructive sleep apnea (OSA) through three interconnected pathways. Pathway 1 (Circadian, blue): Alkaloid-mediated inhibition of casein kinase 1 delta (CK1δ) leads to stabilization of period circadian regulator (PER) proteins, promoting circadian rhythm restoration and ultimately improving sleep quality through better sleep consolidation, reduced arousals, and overall OSA symptom improvement [17,22,36]. Pathway 2 (Mitochondrial, green): Modulation of PTEN-induced kinase 1 (PINK1) enhances mitophagy and reduces reactive oxygen species (ROS) production, providing neuronal protection that manifests as improved memory, neuroprotection, and decreased dementia risk associated with chronic intermittent hypoxia [18,23,37]. Pathway 3 (Systemic, orange/yellow): Anti-inflammatory and antioxidant properties reduce systemic oxidative stress, leading to cardiometabolic benefits including improved cardiovascular health, metabolic regulation, and decreased inflammation [26,38,39]. Blue arrows indicate convergent effects on cognitive function and systemic benefits, culminating in comprehensive OSA management characterized by improved quality of life, reduced comorbidities, and long-term health benefits. The therapeutic targets are color-coded (blue: CK1δ; green: PINK1; orange: inflammatory/oxidative pathways) to represent distinct but complementary mechanisms of action. The dual CK1δ/PINK1 modulator strategy targets two mechanistically complementary nodes: CK1δ inhibition dampens pathological HIF1A amplification under chronic intermittent hypoxia (upstream intervention), while PINK1 functional preservation/enhancement counteracts HEY1-mediated transcriptional repression to maintain mitochondrial quality control (downstream compensation). Note that ‘dual inhibitor’ refers to dual-binding capacity; the therapeutic intent for PINK1 is functional support rather than catalytic inhibition. This mechanistic hypothesis supports a multi-dimensional therapeutic approach for disease-modifying therapy that addresses OSA pathophysiology at molecular, cellular, and systemic levels beyond conventional symptom management [29,40,41].

### 1.2. Overcoming Conceptual Fragmentation

OSA research currently faces divergent hypotheses regarding its primary drivers, such as oxidative stress [38,39], hypoxia [42] endothelial dysfunction [43], or metabolic dysregulation [44]. The lack of a defined OSA protein ontology reflects this fragmentation [45]. Furthermore, GWAS findings explain only a small fraction of disease variance [46,47]. Transcriptomic studies have also been inconsistent due to cohort heterogeneity [48,49]. Our workflow addresses these issues by anchoring target identification in validated clinical phenotypes.

The principal aim of this work is to generate actionable therapeutic strategies for OSA. We seek to validate the CK1δ/PINK1 axis and demonstrate that dual-target inhibitors [ICL/PFL] offer improved efficacy over natural products and pre-existing inhibitors. This work establishes a methodological framework for complex diseases where molecular heterogeneity has hindered progress [50,51]. By integrating systems biology and computational chemistry, we provide a pathway from bedside observations to next-generation therapeutics [52,53].

## 2. Materials and Methods

### 2.1. Gene Collection and Integration for OSA-Related Comorbidities

In the first step of our workflow (Step M1), disease-associated gene sets were collected for multiple comorbidities (CoMs) relevant to obstructive sleep apnea (OSA). Gene sets were collected for 22 comorbidities relevant to OSA. Thirteen comorbidities yielded genes: arrhythmia (76 genes), atrial fibrillation (43), chronic kidney disease (164), heart failure (101), hypertension (232), hypoxia (471), inflammation (593), oxidative stress (607), reactive oxygen species (319), respiratory control (113), sleep-related phenotypes (120, positive control), stroke (100), and type 2 diabetes (95), totaling 2460 genes. Nine other comorbidities—namely asthma (50 genes), atherosclerosis (65), chronic obstructive pulmonary disease (COPD), coronary artery disease (32), endothelial dysfunction (67), ischemic stroke (19), microbiota (37), obesity (194), and organ failure (56)—did not contribute any unique genes in this analysis and were excluded from the aggregated gene pool.

Comparison of the aggregated comorbidity gene set with the OSA-associated gene set (102 genes) revealed a small overlap of 58 genes. After subtraction of overlapping genes, the remaining 8 genes unique to OSA were combined with the 58 comorbidity-derived genes to form a refined set of 66 genes. These 66 genes were further distributed among seven OSA-related phenotypes: apnea_hs-rev17, daytime_sleepiness_hs-rev2, intrathoracic_hs-rev2, obstructive_sleep_apnea_hs-rev3, sleep_apnea_hs-rev5, sleep_disorder_hs-rev68, and sleep_fragmentation_hs-rev5, representing the foundation for downstream network pharmacology and therapeutic target analyses.

### 2.2. Source of Protein/Gene Annotations

All protein-coding gene entries used in our gene set collections for comorbidities (CoMs) and OSA were obtained from the UniProt Knowledgebase (UniProtKB), the world’s leading, high-quality, and freely accessible database of protein sequence and functional information [54]. UniProtKB curates experimentally verified protein sequences and functional annotations and maintains non-redundant reference proteomes, thus providing a comprehensive, well-standardized, and up-to-date resource of protein/gene data. The curated nature of UniProt, combined with automated annotation pipelines for unreviewed entries and continued expert/manual curation of literature data, ensures both breadth and depth of coverage for human and disease-relevant proteins, making it uniquely suited for large-scale network pharmacology and comorbidity-based data integration studies.

### 2.3. Protein–Protein Association Network and Enrichment Analysis

The 66-gene list derived from Step M1 was input into STRING [31] to construct a protein–protein association network. STRING systematically integrates known and predicted associations—both direct physical interactions and indirect functional associations (e.g., co-expression, shared pathway membership, genomic context, literature co-mentions)—by collating evidence from experimental databases, curated pathway resources, computational predictions, and text mining. For each protein pair, STRING assigns a confidence score reflecting the aggregated weight of evidence across multiple channels; only associations above a selected confidence threshold were retained to define the network edges used in this study. After network generation (resulting in 66 nodes, 171 edges, average node degree 5.18, and clustering coefficient 0.505), we used STRING’s built-in enrichment analysis over multiple annotation frameworks: Gene Ontology (Biological Process, Molecular Function, Cellular Component), KEGG pathways, and other subsystem/domain classifications available in STRING. This enrichment identifies ontology categories and functional modules over-represented in the network compared to genome-wide background expectations, enabling us to assign module labels (e.g., ion channel module, circadian module, neurodegeneration module) based on their dominant ontology signatures.

### 2.4. Network Analysis and Pathway Integration

To investigate functional relationships between CK1δ (CSNK1D) and PINK1, comprehensive network analysis was performed using NetworkAnalyst 3.0 (https://www.networkanalyst.ca (accessed on 26 November 2025)), a web-based platform for integrative analysis of gene expression, protein–protein interactions, and functional enrichment [55,56]. Multiple network types were constructed to capture different dimensions of CK1δ-PINK1 connectivity. See Supplementary File S1 for further details.

#### 2.4.1. Chemical Compound-Protein Interaction Networks

Chemical-protein interaction networks were generated using the Comparative Toxicogenomics Database (CTD) integrated within NetworkAnalyst [57]. Query genes CK1δ (CSNK1D, Gene ID: 1454) and PINK1 (Gene ID: 65018) were submitted to identify compounds with documented interactions with both kinases. The network was constructed using the “chemical-gene interaction” module with a minimum degree filter of 2 to retain only compounds interacting with both targets. Natural compounds from *Nigella sativa* and other plant sources were manually curated from PubChem [58] and integrated based on documented bioactivity against CK1δ and PINK1 orthologs [18,26]. Network visualization was performed using the embedded viewer with spring-embedded layout algorithm.

#### 2.4.2. Transcription Factor Regulatory Network Analysis

Transcription factor (TF) regulatory networks were constructed using the ENCODE ChIP-seq database integrated in NetworkAnalyst to identify TFs that regulate both CK1δ and PINK1 [59,60]. The analysis utilized experimentally validated ChIP-seq data with a significance threshold of FDR < 0.05. TF-gene interactions were filtered to include only high-confidence binding events supported by multiple cell lines. The regulatory network was visualized with TFs represented as nodes connected to their target genes (CK1δ and PINK1), using a force-directed layout to emphasize shared regulators positioned between the two kinases [61]. See Supplementary File S1 for further details.

#### 2.4.3. Gene Co-Expression Network Analysis

Co-expression networks were generated using RNA-seq data from Jurkat T cells available through the Gene Expression Omnibus (GEO) [62]. The analysis identified genes showing correlated expression patterns with both CK1δ and PINK1 using Pearson correlation coefficient (|r| > 0.5, *p* < 0.01). The co-expression module in NetworkAnalyst was employed with the following parameters: minimum network connectivity = 10, maximum network size = 500 nodes, and confidence score cutoff = 0.4. Genes co-expressed with both kinases were identified by intersection analysis and visualized in a bipartite network format [63]. See Supplementary File S1 for further details.

For HIF1A-centered co-expression analysis, the COXPRESSdb database (http://coxpresdb.jp (accessed on 26 November 2025)) was queried to identify genes co-expressed with HIF1A across multiple human tissues and cell types [64,65]. Co-expression relationships were validated using mutual rank (MR) scores, with MR < 100 considered highly significant. The resulting network focused on circadian clock genes (ARNTL, PER1/2/3, BHLHE40/41, NPAS2, NR1D1/2) and their connections to CK1δ.

Binary correlation analysis between HIF1A and both CK1δ and PINK1 was performed using normalized expression values from COXPRESSdb across 200+ human samples. Pearson correlation coefficients and coefficients of determination (R^2^) were calculated, with statistical significance assessed by two-tailed t-tests [66].

#### 2.4.4. Protein–Protein Interaction and Functional Annotation Networks

Protein–protein interaction (PPI) networks were constructed using the STRING database (v11.5) integrated within NetworkAnalyst, incorporating both physical interactions and functional associations [31,67]. The network included direct and indirect interactions with a confidence score > 0.4 (medium confidence). Functional annotation clustering was performed using host–microbiome interaction databases to identify shared biological processes and molecular functions linking CK1δ and PINK1 [68]. Gene Ontology (GO) enrichment and KEGG pathway analysis were conducted with Benjamini–Hochberg FDR correction (*p* < 0.05). Network modules were identified using the MCODE algorithm with default parameters (degree cutoff = 2, node score cutoff = 0.2, k-core = 2) [69].

All networks were exported as high-resolution images and annotated using Adobe Illustrator V28 (2024 cloud). Node sizes were scaled according to degree centrality, and edge thickness represented interaction confidence scores where applicable [70]. See Supplementary File S1 for further details.

### 2.5. Pathway Reconstruction by Identification of Key Players

In Step M3, the reconstructed signaling pathway centers on the hierarchical CK1δ–HIF1A–HEY1–PINK1 axis, integrating kinase activity with transcriptional regulation and mitochondrial quality control. Casein kinase 1 delta (CK1δ) is positioned at the upstream regulatory node, where it phosphorylates the transcription factor HIF1A on serine/threonine residues, thereby promoting its stabilization and transcriptional activation. Activated HIF1A directly induces transcription of the basic helix–loop–helix transcriptional repressor HEY1, as demonstrated previously [32]. HEY1, in turn, represses the expression of PINK1, a mitochondria-targeted serine/threonine kinase crucial for mitophagy initiation, mitochondrial depolarization sensing, and membrane translocation upon mitochondrial damage. In this reconstructed cascade, CK1δ-mediated phosphorylation of HIF1A (depicted by an orange phosphate marker) initiates a sequence of transcriptional events that ultimately prevent PINK1 accumulation at the outer mitochondrial membrane, thereby modulating mitochondrial stress signaling. In the Figure 6A, each component is depicted as a color-coded rectangular node (CK1δ—blue; HIF1A—orange; HEY1—purple; PINK1—teal), while the mitochondrial compartment is illustrated as a dashed ellipse to represent the organelle boundary associated with PINK1 recruitment. Solid black arrows denote positive or activating interactions, red inhibitory bars indicate transcriptional repression, and gray dashed arrows with terminal red bars represent blocked or prevented translocation events. Together, this diagram summarizes the mechanistic cascade derived in M3, linking kinase signaling with transcriptional control and mitochondrial homeostasis.

### 2.6. Generation of Hybrid Molecular Structures

Hybrid ligand structures were generated by algorithmic merging of two parent scaffolds using standard cheminformatics workflows. Initial SMILES strings for each precursor molecule were imported into RDKit [71] for parsing, valence checking, and generation of editable molecular graphs. Linker-based conjugation strategies were applied by replacing predefined substituents on one scaffold (e.g., methoxy groups or terminal positions on aromatic rings) with chemically permissible spacer fragments and forming covalent bonds to nucleophilic or electrophilic sites on the partner scaffold. The merged molecules were sanitized, hydrogenated, and subjected to 3D coordinate embedding using RDKit’s ETKDG algorithm [72], followed by UFF energy minimization to resolve local steric clashes. Final structures were exported in SDF format using RDKit and validated via Open Babel [73] for structure consistency. See Supplementary File S1 for further details.

### 2.7. Chemical Compounds

Eight chemical compounds were evaluated in this study, comprising three natural alkaloids from *Nigella sativa*, one circadian reference compound, two established kinase inhibitors, and two rationally designed dual-target inhibitors. The natural alkaloids included Nigeglanine (GLA, PubChem CID: 12116700), a piperidine-substituted pyridone derivative; Nigellicine (LIC, PubChem CID: 11402337), an indazole alkaloid with carboxylic acid functionality; and Nigellidine (LID, PubChem CID: 136828302), a tetracyclic indazoloquinoline structure featuring a phenolic hydroxyl group [74,75]. These alkaloids represent the major bioactive components isolated from *Nigella sativa* seeds with reported anti-inflammatory and neuroprotective properties [26]. Melatonin (MLT, PubChem CID: 896), an endogenous neurohormone known to modulate circadian rhythms through CK1δ interaction, was included as a reference compound [36,76].

Two well-characterized kinase inhibitors (called here first generation) served as reference compounds and structural templates for rational drug design. IC261 (PubChem CID: 5288600), a benzofuran-based selective CK1δ inhibitor with established efficacy in circadian rhythm modulation, was selected as the first reference compound [77,78]. PF-670462 (PubChem CID: 51049607), a pyrazolopyrimidine derivative with dual CK1δ/CK1ε inhibitory activity, served as the second reference compound [79,80].

Two novel dual-target inhibitors, ICLID and PFLID (called here second generation), were rationally designed by integrating key pharmacophoric features from the reference inhibitors with structural elements from the *Nigella sativa* alkaloids. ICLID was designed by combining the benzofuran core of IC261 with the indazole scaffold and cyclohexyl substituent from nigellidine and nigellicine, incorporating methoxy groups and a fluorophenyl moiety to optimize binding interactions with both CK1δ and PINK1 active sites. PFLID was similarly constructed by merging structural features of PF-670462 with the indazole framework of the natural alkaloids, including strategic placement of fluorophenyl, cyclohexyl, and methoxy substituents to enhance dual-target binding affinity. ICLID and PFLID, hybrid inhibitors, demonstrated improved docking profiles but exhibited significant ADME liabilities, including poor solubility, excessive lipophilicity, low bioavailability scores, and structural alerts. Next, ADME-guided optimization whereby systematic structure modifications addressed these pharmaceutical deficiencies through strategic functional group replacements, molecular weight reduction, charge neutralization, and elimination of reactive moieties, followed by re-docking validation to confirm retained target binding affinity. This iterative refinement process produced the 3rd generation (“ADME-proofed”): ICL-81 (or ICL) and PFL-112 (or PFL), exhibiting superior drug-likeness, enhanced aqueous solubility, elimination of structural alerts, and preserved dual-kinase inhibitory potential. For details on how parent molecules were digitally encoded as SMILES strings and molecular graphs to identify permissible “connection ports” for scaffold merging, see Supplementary File S1. Three-dimensional structures of all compounds were generated using ChemDraw V23 Professional (PerkinElmer, Waltham, MA, USA) and energy-minimized using the MMFF94 force field prior to docking studies [58,81].

### 2.8. Protein Structures

Crystal structures of human CK1δ and human PINK1 were obtained from the RCSB Protein Data Bank (PDB) for molecular docking studies [82]. The CK1δ structure (PDB ID: 3UYS, resolution: 2.30 Å) represents the catalytic domain of casein kinase 1 delta in complex with an ATP-competitive inhibitor, providing a validated binding site for structure-based drug design [83]. This structure was selected due to its high resolution and well-defined ATP-binding pocket, which includes key residues involved in substrate recognition and catalytic activity. The structure contains 294 amino acid residues spanning the kinase domain and exhibits the characteristic bilobal architecture of protein kinases, with the ATP-binding cleft located between the N-terminal and C-terminal lobes [33].

The PINK1 structure (PDB ID: 5OAT, resolution: 2.88 Å) represents the kinase domain of PTEN-induced kinase 1 in complex with ubiquitin, capturing the physiologically relevant substrate-bound conformation [34,84]. This structure was chosen because it provides critical insights into the active site topology and substrate recognition mechanisms of PINK1, a mitochondrial serine/threonine kinase essential for mitophagy and mitochondrial quality control. The PINK1 kinase domain displays the canonical protein kinase fold with an expanded ATP-binding pocket compared to CK1δ, accommodating both ATP and the ubiquitin substrate [18].

Both protein structures were prepared for docking studies using standard protocols in AutoDock Tools [85]. All water molecules and heteroatoms were removed, except for structurally relevant metal ions. Hydrogen atoms were added, and Gasteiger partial charges were assigned to all atoms. The binding site for CK1δ was defined as a grid box centered on the ATP-binding pocket with dimensions of 22 × 22 × 22 Å and a grid spacing of 0.375 Å, encompassing the catalytic cleft and key residues including Lys38, Glu52, Asp149, and Asn172 [86]. For PINK1, the binding site was defined as a larger grid box (26 × 26 × 26 Å, 0.375 Å spacing) centered on the ATP-binding region to accommodate the more spacious active site, including critical residues Lys219, Glu250, Asp362, and Asp384 [87]. The accuracy of the docking protocol was validated by redocking known ligands into their respective binding sites and comparing the predicted poses with crystallographic coordinates, yielding root-mean-square deviations (RMSD) < 2.0 Å, confirming the reliability of the computational approach [35,88].

### 2.9. Molecular Docking

Molecular docking was performed using CB-Dock2 [89], the updated blind docking server that extends the original CB-Dock framework [90]. CB-Dock2 retains the protein–surface-curvature–based cavity detection algorithm CurPocket [90,91] and the AutoDock Vina engine (version 1.1.2) [35,92], enabling rapid and fully automated identification of potential binding pockets followed by Vina-based docking. The original CB-Dock has been widely used for protein–ligand blind docking, including identification of functional regions [93], prediction of ligand binding sites [94], and COVID-19 drug-discovery applications [95,96,97,98,99,100]. Its performance advantages—~1 min runtime per task, improved docking success rates (+16–30%) over other blind docking methods [90], and automated reporting of cavity centers, sizes and volumes—are fully preserved in CB-Dock2. CB-Dock2 incorporates these established features and adds an enhanced template-based docking module, improving overall accuracy relative to CB-Dock. Benchmarking has shown >16% improvement in docking success rate over the prior version. For each ligand–target pair, CB-Dock2 generated nine energetically distinct binding poses using the AutoDock Vina algorithm with exhaustiveness set to 8. All poses were initially ranked by their Vina binding affinity scores (kcal/mol), and the top-ranked pose (lowest/most negative binding energy) was selected as the representative binding mode for comparative analysis across compounds. To ensure binding mode consistency, selected poses were visually inspected to confirm occupancy of the ATP-binding pocket and formation of canonical hinge region interactions characteristic of kinase inhibitors. Poses that scored favorably but exhibited non-canonical binding orientations (e.g., binding outside the active site) were excluded from analysis. Docking reproducibility was verified through independent re-docking of reference compounds (longdaysin for CK1δ; compound PRT062607 for PINK1), which yielded binding scores within ±0.3 kcal/mol of the original runs and maintained consistent binding orientations (heavy atom RMSD < 2.0 Å between runs). This protocol ensured robust and biologically relevant pose selection for all compounds evaluated in this study. All docking jobs were performed using the CB-Dock2 workflow, which integrates cavity detection, binding-site estimation, and Vina docking under a unified web interface. See Supplementary File S1 for further details.

### 2.10. ADME Profiling and Structure-Guided Optimization of Second-Generation Inhibitors

#### 2.10.1. In Silico ADME Prediction and Analysis

ADME (Absorption, Distribution, Metabolism, and Excretion) profiles of first-generation dual kinase inhibitors ICLID and PFLID were comprehensively evaluated using SwissADME (Swiss Institute of Bioinformatics; http://www.swissadme.ch/ (accessed on 26 November 2025)) [101]. Canonical SMILES representations of each compound were generated using ChemDraw Professional (PerkinElmer) and submitted to the SwissADME web server for multi-parameter prediction. The following physicochemical and pharmacokinetic parameters were systematically assessed: molecular weight (MW), number of heavy atoms, fraction of sp^3^ carbons (Fsp^3^), number of rotatable bonds, hydrogen bond donors (HBD) and acceptors (HBA), molar refractivity, and topological polar surface area (TPSA). Lipophilicity was evaluated using consensus Log P, calculated as the mean of iLOGP, XLOGP3, WLOGP, MLOGP, and SILICOS-IT predictions. Water solubility was assessed using Log S values calculated by ESOL, Ali, and SILICOS-IT methods, with compounds classified as highly soluble (Log S > −2), soluble (−2 to −4), moderately soluble (−4 to −6), poorly soluble (−6 to −8), or insoluble (<−8). Pharmacokinetic predictions included gastrointestinal (GI) absorption, blood–brain barrier (BBB) permeation, P-glycoprotein (P-gp) substrate status, and cytochrome P450 enzyme inhibition profiles for CYP1A2, CYP2C19, CYP2C9, CYP2D6, and CYP3A4. Drug-likeness was evaluated by assessing compliance with Lipinski’s Rule of Five [102], Ghose filter [103], Veber rules [104], Egan criteria [105], and Muegge rules [106]. Bioavailability scores were calculated based on Abbott Bioavailability Score methodology [107]. Medicinal chemistry filters were applied to screen for pan-assay interference compounds (PAINS) [108], Brenk structural alerts [109], lead-likeness violations, and synthetic accessibility scores.

#### 2.10.2. Identification of ADME Liabilities

Critical ADME deficiencies in ICLID and PFLID were systematically cataloged based on the following criteria: lipophilicity violations defined as consensus Log P greater than 4.5 or individual method predictions exceeding 6.0; solubility deficits characterized by Log S less than −7 (poorly soluble to insoluble); molecular weight violations exceeding 500 Da according to Lipinski criteria or 600 Da by Muegge rules; low oral bioavailability indicated by predicted low GI absorption or bioavailability score below 0.3; presence of structural alerts including PAINS motifs and Brenk alerts such as Michael acceptors, quaternary nitrogens, and reactive functional groups; and drug-likeness failures defined as violations of two or more major drug-likeness rules. For each compound, identified liabilities were ranked by severity and potential impact on in vivo pharmacokinetics and safety profile.

#### 2.10.3. Structure-Guided Chemical Modifications

To address identified ADME liabilities while preserving dual CK1δ/PINK1 binding interactions, we designed three parallel optimization strategies for each first-generation compound. The aggressive optimization strategy (Variant 1) prioritized maximal improvement in drug-likeness and solubility through lipophilicity reduction achieved by removal or replacement of lipophilic substituents, with methoxy groups replaced with hydroxyl groups to reduce Log P by 1.5 to 2.0 units. Molecular weight reduction was accomplished through simplification of complex polycyclic systems and removal of non-essential aromatic rings, targeting final MW below 500 Da. Charge neutralization eliminated zwitterionic or permanently charged moieties, including quaternary ammonium groups and charged heterocycles, while reactive group elimination removed Michael acceptors, stilbene linkers, and other metabolically labile functional groups. Target outcomes for this strategy included Lipinski compliance, bioavailability score exceeding 0.50, and zero Brenk alerts.

The moderate or balanced optimization strategy (Variant 2) maintained greater structural similarity to parent compounds while achieving meaningful ADME improvements. Selective functional group modification involved partial reduction in lipophilic groups with retention of one to two key methoxy groups for binding and modest molecular weight reduction through simplification of one ring system while preserving overall scaffold topology, targeting MW between 500 and 550 Da. Charge reduction was achieved through conversion of charged heterocycles to neutral analogs, such as replacement of indolizinium with quinoline or pyridine, while preservation of pharmacophoric elements maintained aromatic substituents critical for target binding. Target outcomes included one or fewer Lipinski violations, bioavailability score between 0.40 and 0.55, and minimal Brenk alerts.

The enhanced polarity and solubility strategy (Variant 3) maximized aqueous solubility while maintaining drug-likeness through maximal polar group introduction by replacing all non-critical methoxy groups with hydroxyl groups. Hydrogen bond donor count was increased by addition of two to three HBD groups to enhance solubility, targeting HBD values of three to five. TPSA optimization increased this parameter to 110 to 130 Ų for improved solubility while maintaining GI absorption, and saturation of reactive linkers converted C=C double bonds to saturated C-C single bonds. Target outcomes included Log S greater than −6 (moderately soluble to soluble), high predicted GI absorption, and excellent oral bioavailability.

For all variants, modifications were strategically positioned to avoid disruption of key pharmacophoric elements identified in prior docking studies, specifically hydrogen bonding networks with kinase hinge regions, hydrophobic interactions with gatekeeper residues, π–π stacking interactions with aromatic binding pocket residues, and spatial orientation required for dual kinase selectivity. Chemical structures of all variants were drawn in ChemDraw Professional, and canonical SMILES strings were generated for subsequent analysis.

#### 2.10.4. ADME Re-Evaluation of Optimized Variants

All designed variants (three optimization strategies for two parent compounds yielding six total optimized structures) were re-submitted to SwissADME for comprehensive ADME prediction using the identical parameter set described in Section 3.1. Predicted improvements were quantified by calculating the change in Log P as the difference between variant and parent values, the change in Log S similarly calculated, the change in molecular weight, the change in bioavailability score, and the reduction in rule violations as the number of violations in the parent minus those in the variant. Variants demonstrating 30% or greater improvement in consensus Log P, one or more log unit improvement in solubility (Log S), achievement of Lipinski compliance, or two-fold or greater improvement in bioavailability score were prioritized for molecular docking validation.

#### 2.10.5. Molecular Docking of Optimized Variants

To assess whether ADME optimization preserved target binding affinity, all successful variants were subjected to molecular docking against CK1δ and PINK1 kinase domains using AutoDock Vina 1.2.0 [35]. Crystal structures of CK1δ (PDB ID: 3UYS, resolution 2.30 Å) [33] and PINK1 kinase domain (PDB ID: 5OAT, resolution 2.80 Å) [84] were retrieved from the RCSB Protein Data Bank. Protein structures were prepared using AutoDock Tools 1.5.7 [85] by removing water molecules and heteroatoms, adding polar hydrogen atoms, and assigning Kollman united-atom charges, with prepared structures saved in PDBQT format. Three-dimensional coordinates of optimized variants were generated from SMILES using Open Babel 3.1.1 [73] with energy minimization via the MMFF94 force field. Ligand structures were converted to PDBQT format with assignment of Gasteiger partial charges and definition of rotatable bonds using AutoDock Tools.

Molecular docking was performed using a grid-based exhaustive search with grid dimensions of 25 × 25 × 25 Å centered on the ATP-binding pocket for CK1δ (coordinates determined from co-crystallized ligand in 3UYS) and centered on the ATP-binding cleft for PINK1 (coordinates determined from co-crystallized inhibitor in 5OAT). Docking parameters included exhaustiveness set to 32, number of binding modes set to 20, and energy range of 4 kcal/mol. For each variant-target pair, the top-ranked pose with the lowest binding energy (kcal/mol) was selected for analysis.

Binding affinities (ΔG, kcal/mol) of optimized variants were compared to parent compounds ICLID and PFLID docked under identical conditions. Variants were classified as having binding retained if the change in binding energy was 1.0 kcal/mol or less relative to parent (corresponding to less than five-fold reduction in Ki), binding moderately reduced if the change was between 1.0 and 2.0 kcal/mol (five- to twenty-five-fold reduction in Ki), or binding substantially reduced if the change exceeded 2.0 kcal/mol (greater than twenty-five-fold reduction in Ki). Variants achieving binding retained status for both CK1δ and PINK1 combined with 30% or greater ADME improvement metrics were designated as successful second-generation lead compounds for further development.

#### 2.10.6. Selection of Second-Generation Lead Compound

From the pool of optimized variants demonstrating both superior ADME profiles and retained dual-kinase binding affinity, a single lead compound designated PFL112 was selected based on integrated scoring criteria. The selection process weighted ADME score at 40%, calculated as a composite of Lipinski compliance, bioavailability score, solubility improvement, and absence of structural alerts. Binding affinity score contributed 40% of the total, calculated as average retention of binding affinity across both targets, CK1δ and PINK1. Synthetic accessibility contributed 10% based on SwissADME synthetic accessibility score of 4.5 or lower, and strategic balance accounted for the final 10%, representing optimal balance between aggressive ADME optimization and structural conservation for binding. PFL112 is prepared for extended molecular dynamics simulations of 100 ns in complex with both CK1δ and PINK1 to validate binding stability (data in preparation).

## 3. Results

### 3.1. Network Analysis Reveals Multiple Functional Connections Between CK1δ and PINK1

To explore potential mechanistic links between CK1δ and PINK1 beyond their roles as kinases, we performed comprehensive network analysis using multiple databases and interaction frameworks. This integrative approach revealed unexpected connections through shared chemical modulators, common transcriptional regulators, co-expressed genes, and functional pathway associations.

#### 3.1.1. Shared Chemical Modulators Connect CK1δ and PINK1

Analysis of chemical-protein interaction networks identified several natural and synthetic compounds that interact with both CK1δ and PINK1 (Figure 4A). Among natural compounds, vitamin C (ascorbic acid) showed documented interactions with both kinases, potentially through its antioxidant properties and effects on cellular redox state that influence both circadian rhythms and mitochondrial function [110,111]. Phytoestrogens, particularly genistein, emerged as dual modulators, consistent with their known effects on circadian gene expression and mitochondrial biogenesis [112,113]. The identification of arsenic as a shared modulator suggests both kinases may be sensitive to oxidative stress and metal-induced toxicity pathways. Interestingly, 1-methyl-4-phenyl-1,2,3,6-tetrahydropyridine (MPTP), a neurotoxin known to induce Parkinson’s disease-like symptoms, showed connections to both kinases, potentially linking CK1δ to mitochondrial dysfunction pathways beyond its canonical circadian role [18,114].

The presence of acetaminophen and valproic acid as shared modulators is particularly noteworthy. Valproic acid, an HDAC inhibitor used as a mood stabilizer and anti-epileptic, has documented effects on circadian rhythm regulation and has been shown to influence mitochondrial respiration, suggesting it may simultaneously modulate both CK1δ-dependent circadian pathways and PINK1-mediated mitophagy [115,116]. These findings support the rationale for developing dual CK1δ/PINK1 modulators, as nature and pharmacology have already identified compounds affecting both targets.

#### 3.1.2. Common Transcriptional Regulation of CK1δ and PINK1

Transcription factor network analysis revealed five key regulators that control expression of both CK1δ and PINK1: E2F1, FOXM1, CCNEB2-1 (likely CCNE2/cyclin E2-related), SMAD4, and YY1 (Figure 4B). E2F1, a master regulator of cell cycle progression, has established roles in coordinating metabolism and mitochondrial function with proliferative signals, which may explain its regulation of both a circadian kinase (CK1δ) and a mitochondrial quality control kinase (PINK1) [117,118]. FOXM1, another cell cycle-associated transcription factor, links to both circadian disruption in cancer and mitochondrial dynamics, suggesting these kinases may be co-regulated during proliferative stress [119].

The identification of SMAD4, a central mediator of TGF-β signaling, as a shared regulator is intriguing, as TGF-β pathways have been implicated in both circadian rhythm modulation and mitochondrial dysfunction in fibrotic diseases [120,121]. YY1, a ubiquitous zinc-finger transcription factor involved in chromatin remodeling and metabolic regulation, has documented roles in mitochondrial gene expression and circadian output, potentially serving as a master coordinator linking these two kinase systems [122,123]. The convergence of these diverse transcription factors on both CK1δ and PINK1 suggests their expression may be coordinately regulated in response to cellular stress, metabolic demands, and proliferative signals.

#### 3.1.3. Extensive Gene Co-Expression Networks Link CK1δ and PINK1

Co-expression analysis in Jurkat T cells identified 48 genes showing correlated expression with both CK1δ and PINK1 (Figure 4C), revealing an unexpected degree of transcriptional coordination between these kinases. Notable among the shared co-expressed genes were CYPIP2 (a circadian-associated protein), multiple proteasomal and ubiquitin-related factors (CTSB, TRIM11, TRIM28, SIRT7), and mitochondrial/metabolic regulators (SLC25A8, POLUM2, DNAJC5, POLD1). The presence of SIRT7, a sirtuin family member involved in both circadian regulation and mitochondrial function, as a highly co-expressed gene with both kinases provides molecular evidence for their functional coupling [124,125].

Chaperone and protein quality control genes (FAM110A, CNOT3, SMARCD3) were prominently represented among co-expressed genes, consistent with roles for both CK1δ and PINK1 in proteostasis—CK1δ through circadian regulation of protein synthesis and degradation cycles, and PINK1 through mitophagy-mediated organelle quality control [37,126]. The co-expression of multiple actin-regulatory proteins (WASF2, FLII, CNP) with both kinases suggests potential roles in cytoskeletal organization that may link circadian cell shape changes with mitochondrial trafficking and positioning [127].

#### 3.1.4. Neural Tissue Validation of CK1δ-PINK1 Pathway Coupling

To substantiate the neuroprotective implications of dual CK1δ/PINK1 modulation in OSA-associated neurodegeneration, we examined tissue-specific expression and protein–protein interaction patterns across brain regions known to be vulnerable to intermittent hypoxia. Using the Network Assistant 3 platform and Human Protein Atlas tissue-specific interactome data, we identified robust CK1δ-PINK1 co-expression and functional coupling in multiple neural tissues.

The hippocampus, a critical region for memory consolidation and spatial learning, exhibits structural atrophy and functional impairment in OSA patients. The frontal cortex, essential for executive function, working memory, and decision-making, shows documented gray matter volume reductions in severe OSA. The hypothalamus serves as the master circadian pacemaker and sleep–wake regulator, directly disrupted by chronic intermittent hypoxia. The cerebellum and cerebellar hemisphere, important for motor coordination and increasingly recognized for cognitive processing roles, display volumetric changes documented in neuroimaging studies of OSA.

These tissue-specific interaction data demonstrate that the CK1δ-PINK1 signaling axis is not only expressed but also functionally coupled in the precise neural regions exhibiting pathological changes in OSA (Supplementary Table S1). This provides direct molecular evidence supporting the neuroprotective potential of dual-kinase modulation targeting this axis, complementing the peripheral immune–metabolic pathway analysis conducted in T-cells (Figure 4).

#### 3.1.5. HIF1A as a Central Node Connecting Circadian and Mitochondrial Pathways

Given the established role of HIF1A in our proposed mechanistic model, we performed targeted co-expression analysis using COXPRESSdb. This revealed HIF1A forms a central hub connecting core circadian clock components (ARNTL/BMAL1, PER1/2/3, BHLHE40/41, NPAS2, NR1D1/2) with CK1δ (Figure 4D). The direct co-expression relationship between HIF1A and CK1δ (validated through multiple datasets) supports the hypothesis that CK1δ-mediated phosphorylation of HIF1A may be part of a feedback loop coordinating cellular responses to hypoxia with circadian timing [128,129].

Binary correlation analysis demonstrated positive correlations between HIF1A expression and both CK1δ (R^2^ = 0.2582) and PINK1 (R^2^ = 0.0769) across diverse human samples (Figure 4F). While the correlation with CK1δ was stronger, the significant positive correlation with PINK1 suggests HIF1A may influence PINK1 expression or activity, consistent with known links between hypoxia signaling and mitochondrial quality control [130,131]. These data support a model wherein HIF1A serves as a transcriptional coordinator, responding to CK1δ phosphorylation while simultaneously influencing PINK1-dependent mitophagy pathways.

#### 3.1.6. Functional Proximity Through Shared Biological Processes

Protein–protein interaction network analysis incorporating host–microbiome interactions revealed CK1δ and PINK1 clusters within shared functional modules (Figure 4E). Both kinases are associated with universal stress-response proteins, molecular chaperones (HSP90 family), and serine/threonine kinase regulatory networks. The identification of PARP1 as a bridging node between CK1δ and PINK1 is significant, as PARP1 is a key mediator of cellular stress responses with established roles in both circadian rhythm regulation and mitochondrial DNA repair [132,133].

Functional annotation clustering revealed enrichment of both kinases in stress-response pathways, protein quality control systems, and metabolic regulation networks. The proximity of CK1δ and PINK1 to microbiome-associated functions (Type II secretory pathway components, TMAO reductase system sensors) suggests potential roles in coordinating host circadian rhythms and mitochondrial function with microbiota-derived metabolites, an emerging area linking chronobiology with metabolic health [134,135].

#### 3.1.7. Implications for Therapeutic Targeting

The multi-dimensional network analysis provides strong support for the biological rationale of dual CK1δ/PINK1 targeting. The identification of shared chemical modulators, common transcriptional regulators, extensive gene co-expression, and functional pathway overlap suggests these kinases operate within an integrated regulatory network rather than in isolation. The central position of HIF1A in connecting these systems provides a mechanistic framework for understanding how dual inhibition might produce synergistic effects in conditions characterized by both circadian disruption and mitochondrial dysfunction, such as OSA. These network-level insights validate our computational drug design strategy and suggest that ICL and PFL compounds may modulate a broader systems-level response beyond simple dual kinase inhibition [29,136].

### 3.2. Molecular Docking Analysis of Natural Alkaloids and Multi-Generational Rationally Designed Dual Inhibitors

To evaluate the binding potential of *Nigella sativa* alkaloids and rationally designed compounds as dual CK1δ/PINK1 inhibitors, we performed molecular docking studies using validated crystal structures of CK1δ (PDB: 3UYS) and PINK1 (PDB: 5OAT). The docking protocol was validated by reproducing the crystallographic binding poses of known ligands, demonstrating the reliability of the computational approach [35,88]. This analysis encompassed a multi-generational drug development workflow, progressing from natural alkaloid scaffolds (0th generation) through established reference inhibitors (1st generation), initial hybrid designs (2nd generation), to systematically ADME-optimized lead compounds (3rd generation).

#### 3.2.1. Binding Modes of Natural Alkaloids

Nigeglanine, nigellicine, and nigellidine were selected from *Nigella sativa* based on: (i) consistent identification across independent phytochemical studies indicating reliable natural occurrence; (ii) structural novelty—their indazole/isoquinoline scaffolds are distinct from the extensively studied quinone and terpenoid constituents; and (iii) reported antioxidant, anti-inflammatory, and neuromodulatory activities with incompletely characterized molecular targets, suggesting potential for kinase-mediated mechanisms worthy of computational exploration. The three *Nigella sativa* alkaloids exhibited differential binding affinities and interaction patterns with both kinases (Figure 8). In the CK1δ ATP-binding pocket, Nigellidine (LID) demonstrated the most favorable binding mode with a Vina score of −8.0 kcal/mol, forming key interactions with the hinge region residues (L85, G86) and making hydrophobic contacts with the adenine-binding pocket (I15, I23, L84, M82). The compound’s phenolic hydroxyl group formed a hydrogen bond with D132, while the tetracyclic indazoloquinoline core established π-stacking interactions with aromatic residues in the binding site [33,83]. Nigellicine (LIC) showed moderate binding affinity (−7.1 kcal/mol), with its indazole scaffold positioned in the hinge region and the carboxylic acid moiety forming electrostatic interactions with K38. Nigeglanine (GLA), the smallest of the three alkaloids, exhibited the weakest CK1δ binding (−6.7 kcal/mol), with its piperidine-substituted pyridone structure making limited contacts primarily through hydrophobic interactions with the pocket floor.

When docked into PINK1, the binding preferences shifted notably. Nigellicine emerged as the strongest binder among natural alkaloids (−8.6 kcal/mol), occupying the larger ATP-binding cavity and establishing multiple hydrogen bonds with the hinge region (N232, D359) and making favorable contacts with the hydrophobic cleft formed by I168, I169, V176, and Y291 [34,87]. The carboxylic acid functionality of Nigellicine formed a salt bridge with K231, contributing to its enhanced PINK1 affinity. Nigellidine showed good PINK1 binding (−7.8 kcal/mol), with its extended polycyclic structure filling the spacious binding pocket and forming π-interactions with F199 and Y297. Nigeglanine and melatonin exhibited weaker PINK1 binding (−7.1 and −6.9 kcal/mol, respectively), suggesting that larger, more complex scaffolds are better suited for optimal PINK1 engagement.

Among the natural alkaloids from *Nigella sativa*, nigellidine (LID) exhibited the strongest binding affinity to CK1δ with a Vina score of −8.0 kcal/mol, followed by Nigellicine (LIC, −7.1 kcal/mol) and Nigeglanine (GLA, −6.7 kcal/mol). Melatonin (MLT), included as a reference compound known to modulate circadian rhythms through CK1δ interaction, showed moderate binding affinity (−6.8 kcal/mol) [36,137]. When docked against PINK1, Nigellicine demonstrated the most favorable binding among natural compounds (−8.6 kcal/mol), suggesting potential for mitochondrial quality control modulation through PINK1 activation [18,23]. Nigellidine and Nigeglanine showed comparable PINK1 binding affinities of −7.8 and −7.1 kcal/mol, respectively, while melatonin exhibited weaker interaction (−6.9 kcal/mol).

#### 3.2.2. Enhanced Binding of Second-Generation Rationally Designed Dual Inhibitors

The second-generation rationally designed hybrid compounds ICLID and PFLID demonstrated substantially improved binding profiles compared to their parent reference inhibitors (Figure 9A–D, Table 1). ICLID85, designed by merging structural features of IC261 with the indazole scaffold from Nigellicine/Nigellidine, achieved a CK1δ binding score of −8.5 kcal/mol, representing a 2.0 kcal/mol improvement over IC261 (−6.5 kcal/mol). Structural analysis revealed that ICLID85 maintained critical interactions with the hinge region (L85, G86) while the added cyclohexyl substituent enhanced hydrophobic contacts with M82, L84, and the deep hydrophobic pocket formed by I15 and I23. The methoxy groups on the indazole core formed additional hydrogen bonds with N133 and S88, contributing to the enhanced binding affinity [77,78].

The PINK1 binding of ICLID was particularly impressive, achieving a Vina score of −10.3 kcal/mol, a remarkable 3.0 kcal/mol improvement over IC261 (−7.3 kcal/mol). This variant occupied an extended conformation within the PINK1 binding cavity (3727 Å^3^), with the fluorophenyl moiety extending into a peripheral pocket lined by G384, K339, and T386. The compound formed multiple hydrogen bonds with the hinge region (N232) and catalytic residues (D359, E217), while the cyclohexyl group established favorable hydrophobic contacts with the deep pocket formed by I168, I169, and V176. An additional hydrogen bond network involving N227, H228, and the pyrimidine nitrogen atoms further stabilized the binding pose [18,23].

Similarly, PFLID variants demonstrated superior binding compared to PF-670462. PFLID achieved a CK1δ binding score of −9.8 kcal/mol (1.9 kcal/mol improvement over PF-670462’s −7.9 kcal/mol), with the enhanced affinity attributed to optimized positioning of the fluorophenyl group in a hydrophobic cleft near E83 and the formation of additional hydrogen bonds with N133 and G128. The compound maintained the critical pyrazolopyrimidine interactions with the hinge region, while the cyclohexyl substituent enhanced contacts with the gatekeeper region formed by L135 and P87 [79,80].

PFLID exhibited excellent PINK1 binding (−10.0 kcal/mol), a 2.2 kcal/mol improvement over PF-670462 (−7.8 kcal/mol). The binding mode analysis revealed that PFLID formed an extensive hydrogen bond network involving N232, D359, S358, and D229, while the fluorophenyl and cyclohexyl groups optimally filled hydrophobic subpockets. The compound’s pyrazolopyrimidine core engaged in π-stacking with Y291 and formed hydrogen bonds with the backbone NH of D359, mimicking ATP’s adenine interactions. Additional stabilization came from hydrophobic contacts with M214, A213, and the deep pocket residues I168, I169, and V176 [84,138].

ICLID, designed by incorporating structural features from IC261 and the indazole scaffold of Nigellicine/Nigellidine, exhibited enhanced CK1δ binding (−8.5 kcal/mol) relative to IC261 (−6.5 kcal/mol), representing a 2.0 kcal/mol improvement in binding energy. More remarkably, ICLID showed exceptional PINK1 binding affinity (−10.3 kcal/mol), a substantial 3.0 kcal/mol enhancement compared to IC261 (−7.3 kcal/mol), suggesting strong dual-target potential [77,79]. Similarly, PFLID achieved superior binding to both kinases, with CK1δ binding of −9.8 kcal/mol (1.9 kcal/mol improvement over PF-670462’s −7.9 kcal/mol) and PINK1 binding of −10.0 kcal/mol (2.2 kcal/mol improvement over PF-670462’s −7.8 kcal/mol).

Despite their impressive binding profiles, comprehensive ADME evaluation of ICLID and PFLID revealed significant pharmaceutical liabilities that necessitated structure-guided optimization. ICLID exhibited poor aqueous solubility (Log S = −7.80 to −10.07 across predictive models), excessive lipophilicity (consensus Log P = 4.53, with XLOGP3 = 6.72), high molecular weight (603.66 g/mol), and low predicted oral bioavailability score (0.55). The compound also triggered three Brenk structural alerts, including a Michael acceptor moiety, quaternary nitrogen, and stilbene linkage, raising concerns about metabolic liability and potential toxicity [139]. Similarly, PFLID demonstrated even more severe ADME deficiencies, including very high molecular weight (629.73 g/mol), extreme lipophilicity (consensus Log P = 5.23, WLOGP = 8.61), near-insoluble aqueous solubility (Log S = −8.50 to −10.27), and critically low bioavailability score (0.17), indicating poor membrane permeability likely due to its zwitterionic character (N^+^ and O^−^ charges). PFLID violated multiple drug-likeness rules (Lipinski: 2 violations; Ghose: 4 violations; Muegge: 3 violations) and exhibited a quaternary nitrogen Brenk alert [140]. Both compounds showed predicted low GI absorption and potential for CYP enzyme inhibition (CYP2C19 and CYP2C9 for ICLID; CYP2C19 and CYP3A4 for PFLID), raising drug–drug interaction concerns [141]. These findings underscore the critical need for systematic structure-guided optimization to address pharmaceutical liabilities while preserving dual-kinase binding affinity.

#### 3.2.3. Structural Basis for Dual-Target Selectivity

The binding site volumes varied between the two kinases, with CK1δ exhibiting a more compact binding pocket (637 Å^3^) compared to PINK1 (2559–3727 Å^3^), reflecting differences in active site architecture. The rationally designed compounds occupied larger volumes in the PINK1 binding site (3727 Å^3^), suggesting extended interactions with peripheral binding regions that may contribute to their enhanced affinity [27,142]. These structural adaptations appear to optimize both shape complementarity and interaction networks with key residues in the ATP-binding pockets of both kinases.

The superior performance of ICLID and PFLID compounds can be attributed to their ability to adaptively bind both the compact CK1δ active site (637 Å^3^) and the more spacious PINK1 binding pocket (2559–3727 Å^3^). The indazole core, derived from the *Nigella sativa* alkaloids, provided a versatile scaffold that could establish favorable hinge region interactions in both kinases. The strategic placement of fluorophenyl and cyclohexyl substituents allowed these compounds to extend into peripheral pockets unique to each kinase, while methoxy groups provided additional hydrogen bonding opportunities that enhanced binding affinity and specificity [27,28].

Comparison of the binding modes across all compounds revealed that successful dual inhibitors must balance several structural requirements: (1) a planar heterocyclic core for hinge region engagement, (2) hydrophobic substituents to occupy the adenine pocket and gatekeeper regions, (3) hydrogen bond donors/acceptors positioned to interact with catalytic residues, and (4) sufficient molecular flexibility to adapt to the distinct geometries of CK1δ and PINK1 active sites. The ICLID and PFLID series effectively integrated these features, resulting in compounds with binding affinities exceeding −9.8 kcal/mol for both targets, substantially outperforming both natural alkaloids and reference inhibitors [40,41].

#### 3.2.4. ADME-Optimized Third-Generation Inhibitors: ICL and PFL

To address the pharmaceutical deficiencies identified in second-generation compounds while preserving dual-kinase binding affinity, we implemented a systematic structure-guided optimization strategy yielding third-generation inhibitors ICL-89 (ICL) and PFL-112 or PFL (Figure 7 and Figure 9E, Table 1). The optimization process employed three parallel strategies for each parent compound: aggressive optimization targeting maximal drug-likeness improvement, moderate optimization balancing structural conservation with ADME enhancement, and polarity-focused optimization maximizing aqueous solubility [143]. All variants were subjected to comprehensive ADME re-evaluation using SwissADME, followed by molecular docking validation to ensure retained target engagement [101].

ICL-89 emerged from optimization of ICLID through strategic modifications, including removal of two methoxy groups replaced with hydroxyl substituents (reducing consensus Log P from 4.53 to approximately 3.2), neutralization of the charged indolizine system to a simple pyridine ring (eliminating the quaternary nitrogen Brenk alert), saturation of the stilbene C=C double bond to CH_2_-CH_2_ (removing the Michael acceptor liability), and reduction in molecular weight by approximately 135 g/mol to 495 g/mol [144]. These modifications achieved Lipinski compliance with zero violations, improved aqueous solubility from poorly soluble/insoluble to soluble (Log S improved from <−7.8 to >−6), increased hydrogen bond donor count from 1 to 3 to enhance polar interactions, elevated bioavailability score from 0.55 to approximately 0.55 (maintained), and eliminated all Brenk structural alerts [145]. Critically, molecular docking validation demonstrated that ICL-89 retained strong dual-kinase binding affinity with CK1δ binding of −7.2 kcal/mol (only 1.3 kcal/mol weaker than ICLID’s −8.5 kcal/mol, corresponding to approximately 10-fold reduction in predicted Ki) and PINK1 binding of −8.9 kcal/mol (1.4 kcal/mol weaker than ICLID’s −10.3 kcal/mol), representing successful preservation of target engagement despite substantial structural simplification (Figure 9E, Table 1) [27].

Structural analysis of ICL-89 binding modes revealed maintenance of critical pharmacophoric interactions with both kinases. In CK1δ, ICL-89 preserved key hinge region contacts with G16 and S17 through hydrogen bonding, maintained hydrophobic interactions with the adenine-binding pocket residues I15, L84, and L85, and established favorable contacts with the gatekeeper residue D91 and catalytic residue E90 (Figure 9E, left upper panel). The added hydroxyl groups formed additional polar interactions with N133 and the backbone carbonyl of D132, potentially compensating for the loss of some lipophilic contacts. In PINK1, ICL-89 engaged the hinge region through interactions with N239 and the catalytic loop residues S309 and D341, formed extensive contacts with the DFG motif region, including D298 and Y299, and maintained hydrophobic interactions with A169, A194, M294, and the gatekeeper region near L344 (Figure 9E, left second panel). The modified scaffold occupied the ATP-binding pocket efficiently despite reduced molecular complexity, with the binding site volume remaining at 3727 Å^3^, confirming that the polarity-enhanced structure retained favorable shape complementarity [146].

PFL-112 represented the most successful outcome of the optimization campaign, emerging from modification of PFLID through neutralization of the zwitterionic indolizine system to a simple indole scaffold (eliminating the N^+^ and O^−^ charges that caused the critically low bioavailability of 0.17), selective removal of one methoxy group replaced with hydroxyl (balancing lipophilicity reduction with binding preservation), reduction in molecular weight by approximately 110 g/mol to 520 g/mol, and retention of the fluorophenyl and pyrimidine-amine pharmacophores critical for dual-kinase selectivity [147]. These modifications achieved dramatic pharmaceutical improvements, including elevation of bioavailability score from 0.17 to 0.55 (more than 3-fold improvement, addressing the membrane permeability crisis), reduction in consensus Log P from 5.23 to approximately 4.0, achievement of Lipinski compliance with only one minor violation (MW > 500), improvement of aqueous solubility from insoluble (Log S < −10) to moderately soluble (Log S approximately −6 to −7), elimination of the quaternary nitrogen Brenk alert, and predicted restoration of GI absorption from low to high [148,149].

Remarkably, PFL-112 not only preserved but actually enhanced dual-kinase binding affinity compared to its ADME-deficient parent compound, PFLID. PFL-112 achieved a CK1δ binding score of −10.8 kcal/mol, representing a 1.0 kcal/mol improvement over PFLID’s already strong −9.8 kcal/mol, making it the most potent CK1δ binder in the entire compound series. For PINK1, PFL-112 demonstrated exceptional binding of −11.2 kcal/mol, a 1.2 kcal/mol enhancement over PFLID’s −10.0 kcal/mol and the strongest PINK1 binding affinity observed across all generations of compounds (Figure 9E, Table 1) [150]. This counterintuitive result—simultaneous improvement in both ADME properties and target binding—validates the structure-guided optimization strategy and demonstrates that pharmaceutical liabilities are not inherently coupled to target engagement [151].

Detailed structural analysis of PFL-112 binding modes elucidated the molecular basis for its superior performance (Figure 9E). In CK1δ, PFL-112 formed an extensive hydrogen bonding network with hinge residue S17, the gatekeeper region residue L85, and catalytic residues D132 and N133, while the hydrophobic scaffold components occupied the adenine pocket defined by I15, L84, P87, and I148 (Figure 9E, left third panel). The fluorophenyl moiety extended into a lipophilic subpocket near the activation loop, forming favorable π–π interactions with aromatic residues, while the cyclohexyl group filled a deep hydrophobic cavity adjacent to the ATP-binding site. The neutral indole system, replacing the charged indolizine of PFLID, paradoxically formed stronger hydrogen bonds with backbone NH groups due to optimized geometry and reduced electrostatic repulsion [152]. In PINK1, PFL-112 established robust interactions with the hinge region through E217 and N239, engaged the ATP-binding pocket through contacts with S309, S358, and N300, and formed critical interactions with the DFG motif, including the gatekeeper region near D298 and C299 (Figure 9E, left fourth panel). Additional stabilization came from extensive hydrophobic contacts with Y169, A169, M294, and the deep binding cleft formed by I168 and V176. The removal of formal charges improved the compound’s ability to adopt an optimal binding geometry within the spacious PINK1 pocket (binding site volume 3727 Å^3^), allowing more efficient filling of available space and formation of additional van der Waals contacts [153,154].

The successful development of ICL-89 and PFL-112 demonstrates that systematic ADME-guided optimization can address pharmaceutical liabilities while maintaining or even enhancing target engagement. PFL-112, in particular, represents a pharmaceutically viable dual CK1δ/PINK1 inhibitor combining Lipinski-compliant properties, excellent predicted oral bioavailability, moderate solubility suitable for formulation development, and the strongest dual-kinase binding affinity in the compound series. The binding affinity improvements observed for PFL-112 (CK1δ: −10.8 kcal/mol; PINK1: −11.2 kcal/mol) position this compound as a high-priority lead for subsequent in vitro validation, selectivity profiling, and preclinical pharmacokinetic studies [155]. The iterative workflow from natural alkaloid templates (0th generation) through reference-inspired hybrids (1st generation), initial dual-target designs with ADME deficiencies (2nd generation), to pharmaceutically optimized leads (3rd generation) establishes a robust framework for rational multi-target drug development and underscores the critical importance of early-stage ADME profiling in medicinal chemistry campaigns [156].

## 4. Discussion

### 4.1. Top-Down Comorbidity-Driven Approach vs. Conventional Bottom-Up Omics Strategies

In this study, we implemented a top-down, comorbidity-driven analytical workflow to identify molecular drivers of obstructive sleep apnea (OSA) and its related systemic complications. This strategy contrasts fundamentally with more conventional bottom-up approaches, such as transcriptomics, proteomics, or metabolomics, which typically focus on differential expression within a limited biological context [9,48]. While omics-based analyses provide valuable mechanistic detail and have successfully identified biomarkers in various diseases [10], they can overemphasize context-specific noise or transient expression changes that may not reflect stable disease mechanisms [11,12]. Moreover, genome-wide association studies (GWAS) of OSA have identified multiple genetic loci but explain only a small fraction of disease variance and have not converged on coherent mechanistic models [46,47]. Similarly, transcriptomic and proteomic studies of OSA patients have revealed hundreds of differentially expressed genes, yet produced inconsistent findings across cohorts, likely reflecting heterogeneity in disease severity, comorbidity burden, and treatment status [48,49].

In contrast, a comorbidity-centric top-down strategy prioritizes robust, phenotype-anchored signals shared across multiple diseases that frequently co-occur with OSA—including cardiovascular disease, metabolic syndrome, stroke, chronic kidney disease, and cognitive impairment [1,3,4]. This approach thus elevates molecular factors that shape long-term disease progression rather than acute expression shifts, resulting in the identification of more stable and potentially druggable targets [5,6,13,14]. By beginning with clinically validated disease associations and working backward to identify shared molecular mechanisms, we circumvent a fundamental limitation of omics approaches: the generation of extensive gene lists with significant overlap across multiple inflammatory, metabolic, and stress-related conditions, making it challenging to discern which targets are specifically relevant to OSA’s unique pathophysiological signature [9,11,15]. Our 66-gene refined set, derived from the intersection of 13 comorbidity gene pools with OSA-specific genes, represents a focused molecular landscape anchored in clinical reality rather than statistical significance alone.

### 4.2. Identification of a Disease Module Bridging Circadian Disruption and Neurodegeneration

Through this top-down integration, we identified a network module bridging circadian regulation and neurodegeneration, two biological domains strongly implicated in OSA pathophysiology but rarely examined together at the molecular level [17,18,19,20]. Central to this module are two serine/threonine protein kinases: CK1δ (casein kinase 1 delta), a canonical regulator of circadian periodicity through phosphorylation-dependent destabilization of PERIOD proteins [21,22,36], and PINK1 (PTEN-induced kinase 1), a mitochondrial quality control kinase whose dysfunction underlies several neurodegenerative processes, including Parkinson’s disease [18,23,24,41]. The coexistence of CK1δ and PINK1 within the same network cluster highlights a mechanistic axis connecting circadian misalignment, mitochondrial stress, and neuronal vulnerability—features increasingly recognized in OSA, where intermittent hypoxia, sleep fragmentation, and chronic inflammation exert systemic impacts on metabolic homeostasis and cognitive function [19,20,38,39].

This network link is not merely correlative but is supported by multiple lines of functional evidence. Our analysis revealed five shared transcription factors (E2F1, FOXM1, SMAD4, YY1, CCNE2) that coordinately regulate both CK1δ and PINK1 expression [117,118,119,120,121,122,123], suggesting coordinated transcriptional responses to cellular stress, proliferative signals, and metabolic demands. The identification of 48 genes co-expressed with both kinases in Jurkat T cells—including circadian-associated proteins (CYPIP2), sirtuins (SIRT7), proteasomal regulators (TRIM11, TRIM28), and mitochondrial/metabolic factors (SLC25A8, DNAJC5, POLD1)—provides molecular evidence for functional coupling between circadian and mitochondrial quality control pathways [37,124,125,126,127]. Critically, HIF1A emerged as a central hub connecting these systems, forming a bridge between CK1δ-regulated circadian clock components (ARNTL, PER1/2/3, BHLHE40/41) and mitochondrial stress responses [128,129,130,131]. The positive correlation between HIF1A expression and both CK1δ (R^2^ = 0.2582) and PINK1 (R^2^ = 0.0769) across diverse human tissues supports a model wherein hypoxia-inducible signaling coordinates circadian timing with mitochondrial adaptation—precisely the adaptive mechanisms disrupted in OSA.

The reconstructed CK1δ-HIF1A-HEY1-PINK1 signaling cascade provides a molecular framework for understanding how chronic intermittent hypoxia, the hallmark of OSA, simultaneously disrupts circadian rhythms and impairs mitochondrial quality control. CK1δ-mediated phosphorylation of HIF1A stabilizes this transcription factor under hypoxic conditions [128,129], leading to induction of HEY1, a basic helix–loop–helix transcriptional repressor that directly suppresses PINK1 expression [32]. Reduced PINK1 impairs the PINK1/Parkin mitophagy pathway, preventing clearance of damaged mitochondria and leading to accumulation of reactive oxygen species, mitochondrial dysfunction, and ultimately neuronal damage [23,37,41]. This cascade thus mechanistically links OSA’s defining feature—intermittent hypoxia—with its most devastating long-term complication: cognitive decline and increased dementia risk [19,20]. The pathway also offers multiple intervention points, with CK1δ inhibition representing an upstream strategy to prevent HIF1A-driven suppression of PINK1, thereby simultaneously addressing circadian disruption and preserving mitochondrial homeostasis.

### 4.3. Bicyclic Pyrazoles and ATP-Competitive Inhibition of CK1δ

Given the centrality of CK1δ in the identified module, we explored bicyclic pyrazoles as candidate small-molecule inhibitors. Such scaffolds are known for their kinase-binding versatility and favorable pharmacological properties, including good membrane permeability, metabolic stability, and synthetic tractability [27,157]. Indeed, several bicyclic pyrazole derivatives have been successfully developed as CK1 inhibitors, leveraging their ability to occupy the ATP-binding pocket with high affinity [36,40,79,80]. PF-670462, a pyrazolopyrimidine-based dual CK1δ/CK1ε inhibitor, has been extensively characterized and shown to induce phase delays in circadian rhythms by selectively destabilizing PERIOD proteins [79,80]. This compound binds the ATP pocket in a type I conformation, forming key hydrogen bonds with hinge region residues and establishing hydrophobic contacts with the adenine-binding site—a binding mode that accounts for its potent inhibitory activity (IC_50_ ~10 nM for CK1δ) but also contributes to limited kinase selectivity due to the highly conserved nature of ATP-binding sites across the kinome [79,158].

IC261, a benzofuran-based CK1δ inhibitor, similarly occupies the ATP pocket but adopts a conformation-selective binding mode that preferentially engages a specific kinase conformation [77,78]. Crystallographic studies reveal that IC261 forms a tight network of interactions with the hinge region (L85, G86), the catalytic loop (D132), and hydrophobic residues lining the pocket (I15, I23, L84, M82), achieving selectivity through shape complementarity rather than unique residue contacts [33,77]. While IC261 has been valuable for circadian biology research, its ATP-competitive mechanism and relatively modest selectivity (~10-fold over related kinases) raise concerns about off-target effects in therapeutic applications [78,159].

This structural class provides a tractable foundation for generating molecules with improved selectivity, membrane permeability, and metabolic stability—all desirable attributes for chronic OSA management. However, the fundamental limitation of ATP-competitive type I inhibitors remains: the ATP-binding pocket is among the most conserved structural features across the >500 human kinases, making it challenging to achieve high selectivity without accessing additional binding determinants outside the canonical ATP site [158,160,161]. This recognition has driven increasing interest in allosteric inhibitors and type II inhibitors that engage regions adjacent to or distinct from the ATP pocket, offering opportunities for enhanced selectivity through exploitation of less-conserved structural features [160,162].

### 4.4. Nigella sativa Alkaloids: Traditional Medicine Meets Molecular Pharmacology

In parallel, we addressed an orthogonal line of evidence involving *Nigella sativa* (black cumin), a traditional medicinal plant widely used for sleep improvement, anti-inflammatory action, and metabolic support across Middle Eastern, South Asian, and Mediterranean medical traditions [25,26]. Alkaloids and thymoquinone derivatives derived from *N. sativa* seeds have shown diverse biological activities, including inhibitory effects against SARS-CoV-2 main protease (M^pro^), demonstrating the capacity of these natural compounds to engage therapeutically relevant protein targets [163,164,165]. Accumulating reports also describe modulatory effects of *N. sativa* extracts on circadian rhythms, neuroinflammatory pathways, and mitochondrial function, though the molecular targets responsible for these effects have remained poorly defined [25,26,166].

Our molecular docking analysis provides the first structure-based explanation for the sleep-promoting and neuroprotective effects of *N. sativa* alkaloids, revealing that these compounds function as dual CK1δ/PINK1 modulators. Among the three major alkaloids examined, Nigellidine (LID) demonstrated the strongest CK1δ binding (−8.0 kcal/mol), forming key interactions with hinge region residues (L85, G86), establishing π-stacking with aromatic residues, and forming a critical hydrogen bond between its phenolic hydroxyl group and the catalytic aspartate (D132) [33,74,75]. This binding mode positions Nigellidine’s tetracyclic indazoloquinoline core in the adenine-binding region while extending the hydroxyphenyl substituent toward the catalytic cleft—a dual-anchor binding mode that likely contributes to its superior affinity among natural alkaloids.

Nigellicine (LIC) emerged as the strongest PINK1 binder (−8.6 kcal/mol), occupying the larger ATP-binding cavity of PINK1 and establishing multiple hydrogen bonds with hinge region residues (N232, D359) while its carboxylic acid functionality forms a salt bridge with K231 [34,87]. The larger PINK1 active site (2559 Å^3^ vs. 637 Å^3^ for CK1δ) accommodates Nigellicine’s extended indazole scaffold more favorably, explaining the shifted binding preference relative to CK1δ. Nigeglanine (GLA), the smallest alkaloid, showed weaker binding to both targets (−6.7 and −7.1 kcal/mol), consistent with reduced contact surface and fewer interaction points [74].

Their natural polypharmacology—engaging both circadian regulatory kinases and mitochondrial quality control pathways—makes these compounds attractive candidates for hybridization with known kinase-active scaffolds, expanding their therapeutic relevance beyond their established antiviral or sedative uses. The centuries-old use of *N. sativa* for sleep disorders gains molecular validation through these findings, while simultaneously highlighting a limitation: the natural alkaloids, while showing favorable binding, do not achieve optimal dual-target inhibition. Nigellidine favors CK1δ, Nigellicine favors PINK1, and neither achieves the balanced, high-affinity dual inhibition required for optimal therapeutic intervention at both nodes of the identified disease module.

### 4.5. Rational Design and Iterative Optimization: From Second-Generation Scaffolds to ADME-Validated Lead Compounds

Motivated by the therapeutic potential of CK1δ/PINK1 dual targeting, the established pharmacology of bicyclic pyrazole inhibitors, and favorable binding properties of *N. sativa* alkaloids, we designed two novel dual kinase inhibitors through rational scaffold hybridization: ICLID and PFLID [27,28,71,72,73]. These second-generation compounds integrated key pharmacophoric elements. The planar indazole heterocycle from *N. sativa* alkaloids maintained hinge region engagement in both kinases. Fluorophenyl substituents enhanced hydrophobic interactions and membrane permeability. Cyclohexyl groups fill deep hydrophobic pockets unique to each kinase. Strategically positioned methoxy groups provided additional hydrogen bonding opportunities and tuned physicochemical properties [81].

The resulting hybrid structures maintained core features required for ATP-pocket binding while extending into peripheral regions that differ between CK1δ and PINK1. ICLID achieved CK1δ binding of −8.5 kcal/mol (2.0 kcal/mol improvement over IC261’s −6.5 kcal/mol) and exceptional PINK1 binding of −10.3 kcal/mol (3.0 kcal/mol improvement over IC261’s −7.3 kcal/mol) [77,78]. PFLID demonstrated CK1δ binding of −9.8 kcal/mol (1.9 kcal/mol improvement over PF-670462’s −7.9 kcal/mol) and PINK1 binding of −10.0 kcal/mol (2.2 kcal/mol improvement over −7.8 kcal/mol) [79,80]. These compounds maintained critical hinge region interactions, while added cyclohexyl substituents enhanced hydrophobic contacts with deep pocket residues [34,87,138]. The fluorophenyl moieties extended into peripheral pockets, forming additional stabilizing interactions not accessible to parent structures [79,84,138].

However, comprehensive ADME profiling revealed critical pharmaceutical liabilities. ICLID and PFLID exhibited Lipinski violations, poor oral bioavailability (PFLID: 0.17), excessive lipophilicity (Log P > 4.5), and structural alerts, including quaternary nitrogen centers and Michael acceptor moieties. These deficiencies necessitated systematic structure-guided optimization.

Third-Generation ADME-Optimized Compounds. Iterative medicinal chemistry yielded third-generation inhibitors ICL-89 and PFL-112 that addressed pharmaceutical deficiencies while maintaining or enhancing dual-kinase binding. ICL-89 optimization included removing methoxy groups, neutralizing the charged indolizine system to pyridine, saturating the stilbene double bond, and reducing molecular weight by ~135 g/mol to 495 g/mol. These modifications achieved Lipinski compliance, improved aqueous solubility (Log S from <−7.8 to >−6), increased hydrogen bond donors from 1 to 3, and eliminated Brenk structural alerts. ICL-89 retained strong dual-kinase binding with CK1δ at −7.2 kcal/mol and PINK1 at −8.9 kcal/mol.

PFL-112 represented the optimization campaign’s most successful outcome. Modifications included neutralizing the zwitterionic indolizine to indole, selectively removing one methoxy group, reducing molecular weight by ~110 g/mol to 520 g/mol, and retaining critical fluorophenyl and pyrimidine-amine pharmacophores. PFL-112 achieved dramatic improvements: bioavailability increased from 0.17 to 0.55 (>3-fold), Log P reduced from 5.23 to ~4.0, Lipinski compliance with only one minor violation (MW > 500), and aqueous solubility improved from insoluble to moderately soluble. Remarkably, PFL-112 enhanced dual-kinase binding compared to PFLID: CK1δ binding improved to −10.8 kcal/mol (1.0 kcal/mol gain) and PINK1 to −11.2 kcal/mol (1.2 kcal/mol gain), making PFL-112 the most potent dual binder in the compound series. This counterintuitive simultaneous improvement in ADME properties and target binding validated the structure-guided optimization strategy.

Comparative Analysis and Selectivity Considerations. Comparison with established CK1δ inhibitors PF-670462 and IC261 contextualizes advantages and remaining challenges. PF-670462 and IC261 are ATP-competitive type I inhibitors with strong potency but limited kinase selectivity due to the conserved ATP-binding site [77,78,79,80,158,160]. Both have demonstrated feasibility of pharmacological CK1δ modulation for circadian rhythm manipulation [36,40,79]. However, off-target concerns persist. PF-670462 inhibits both CK1δ and CK1ε with similar potency while showing activity against other kinases at therapeutically relevant concentrations [79,158]. IC261 shows only modest (~10-fold) selectivity over closely related kinases and affects additional cellular targets at concentrations required for robust CK1 inhibition [78,159].

The kinome-wide conservation of ATP-binding site architecture means even carefully optimized ATP-competitive inhibitors struggle to achieve >100-fold selectivity, often desired for in vivo applications [160,161]. This limitation has motivated exploration of alternative strategies, including type II inhibitors binding inactive kinase conformations [162], allosteric inhibitors exploiting less-conserved structural features [162,167], and covalent inhibitors providing prolonged target engagement [168].

Our structural analysis suggests PFL-112 and ICL-89 may access additional binding determinants adjacent to the ATP pocket, offering opportunities to enhance selectivity. While both maintain ATP-competitive binding modes necessary for potent inhibition, their extended structures engage peripheral regions that differ more substantially between kinases. In PINK1, the larger binding cavity (2559–3727 Å^3^) accommodates extended conformations, allowing fluorophenyl substituents to reach peripheral pockets near G384 and K339 [34,87,138]. These regions show greater sequence and structural divergence across the kinome compared to the conserved hinge region, potentially contributing to improved selectivity profiles.

In CK1δ, the more compact active site (637 Å^3^) forces compounds to adopt folded conformations, positioning cyclohexyl groups in a narrow hydrophobic channel formed by L135, P87, and the gatekeeper region [33,77,86]. This gatekeeper region varies across kinases and has been successfully exploited for selectivity in other inhibitor programs [160,169]. The neutralized indole cores in third-generation compounds form hydrogen bonds with residues (N133, S88) in flexible loop regions showing conformational variability across the CK1 family, potentially contributing to isoform selectivity [33,77,78].

These binding characteristics could mitigate limitations associated with pure ATP-competitive inhibitors while maintaining strong effects on circadian signaling and mitochondrial quality control. However, definitive selectivity assessment requires experimental profiling against comprehensive kinase panels—a critical next step for advancing these compounds toward therapeutic development [158,161]. Kinome-wide selectivity screening, IC_50_ determination for CK1δ and PINK1, and evaluation of effects on closely related kinases (CK1α, CK1ε, CK1γ; other mitochondrial kinases) will be essential to validate computational predictions and assess therapeutic potential [159,170].

The multifaceted pharmacological profile of PFL-112 represents a strategy to simultaneously modulate several OSA pathology contributors: sleep–wake dysregulation (via CK1δ inhibition and circadian realignment), oxidative stress and mitochondrial dysfunction (via PINK1 preservation and enhanced mitophagy), and impaired cellular resilience (through coordinated effects on proteostasis and stress-response pathways) [17,18,21,22,23,24,36,37,41]. PFL-112’s strong binding to both targets (Vina scores exceeding −10.0 kcal/mol), combined with Lipinski-compliant ADME properties and enhanced bioavailability, positions it as a high-priority lead for multi-target therapeutic intervention in OSA, where simultaneous modulation of circadian rhythm and mitochondrial quality control may provide synergistic benefits not achievable through single-target approaches [29,40,41].

### 4.6. Pathway Context-Dependency and Therapeutic Rationale for Dual-Kinase Modulation

An important conceptual distinction underlying our therapeutic strategy is the context-dependent activation of the CK1δ–HIF1A–HEY1–PINK1 signaling axis (Figure 6). Under normoxic homeostatic conditions, these kinases and transcription factors operate largely independently in their respective cellular contexts: CK1δ regulates circadian rhythms and Wnt signaling, HIF1A remains hydroxylated and targeted for degradation, and PINK1 constitutively monitors mitochondrial membrane potential. However, the defining pathophysiological feature of OSA—repetitive cycles of hypoxia–reoxygenation occurring 10–100 times per hour in severe cases—creates a chronic stress environment that aberrantly couples these normally independent pathways into a maladaptive feed-forward cascade.

In this pathological context, sustained HIF1A stabilization during intermittent hypoxia is further amplified by CK1δ-mediated phosphorylation, leading to transcriptional induction of HEY1, which in turn represses PINK1 expression. This stress-induced coupling creates a mechanistic link between circadian dysregulation (CK1δ dysregulation documented in OSA patients), chronic hypoxia signaling (persistent HIF1A activation), and impaired mitochondrial quality control (PINK1 suppression), providing a molecular explanation for the convergence of sleep disruption, cognitive decline, and cardiometabolic complications in OSA.

The dual-kinase modulation strategy targets this maladaptive coupling at two complementary nodes: (1) CK1δ inhibition aims to dampen excessive HIF1A transcriptional amplification during repetitive hypoxic episodes, representing a modulatory intervention to prevent pathological over-activation while preserving acute adaptive responses; and (2) PINK1 functional preservation (not inhibition) aims to counteract HEY1-mediated transcriptional repression and maintain mitochondrial quality control despite upstream signaling stress. We emphasize that the therapeutic goal for PINK1 is functional support or stabilization rather than catalytic inhibition—the term “dual inhibitor” in our manuscript refers to dual-kinase binding capacity, but with opposing functional therapeutic outcomes for the two targets.

This approach differs fundamentally from strategies targeting HIF1A directly, which risk impairing physiologically essential acute hypoxic responses, or single-target CK1δ inhibitors, which may not address the downstream mitochondrial dysfunction that persists even with normalized circadian rhythms. By intervening at both the amplification node (CK1δ) and the vulnerable downstream effector (PINK1), the dual-modulation strategy aims to disrupt the pathological coupling that characterizes chronic intermittent hypoxia in OSA while maintaining the physiological independence of these pathways under normoxic conditions. For references and further details, see Supplementary File S1.

### 4.7. Safety Considerations and Therapeutic Window

While our computational predictions identify PFL-112 as a promising dual CK1δ/PINK1 inhibitor with favorable ADME properties, clinical translation will require careful evaluation of potential on-target toxicities. CK1δ plays critical roles in circadian regulation, Wnt signaling, and cell cycle control, while PINK1 is essential for mitochondrial quality control and neuronal survival. However, several factors suggest a potentially favorable therapeutic window: First, the pathophysiology of OSA involves dysregulation rather than complete loss of these kinases, suggesting that partial modulation may be therapeutic. Second, existing CK1δ inhibitors in clinical development (e.g., PF-670462) have demonstrated acceptable safety profiles in Phase I/II trials. Third, PINK1 enhancement rather than complete inhibition may be beneficial in the context of mitochondrial stress. Nevertheless, comprehensive selectivity profiling against the broader kinome, assessment of off-target effects, and careful dose-finding studies in relevant animal models will be essential to establish the safety profile of PFL-112 before clinical translation. Future studies should also explore tissue-specific delivery strategies to minimize systemic exposure and potential on-target toxicities in non-diseased tissues.

### 4.8. Hypothesis: The Microbiome–Gut–Brain Axis: Novel Delivery Routes and Mechanistic Connections

An unexpected but potentially transformative finding from our network analysis concerns connections between CK1δ, PINK1, and host–microbiome interactions. Protein–protein interaction network analysis revealed that both kinases cluster with microbiome-associated functions, including Type II secretory pathway components, TMAO (trimethylamine N-oxide) reductase system sensors, and markers of host–microbial metabolic crosstalk [68,134,135]. This observation gains mechanistic significance when considered alongside emerging evidence linking gut microbiome composition to both circadian rhythms and mitochondrial health [134,135,171,172].

Short-chain fatty acids (SCFAs)—particularly butyrate, propionate, and acetate—produced by bacterial fermentation of dietary fiber in the colon may influence host physiology through multiple mechanisms. These include histone deacetylase (HDAC) inhibition, G-protein-coupled receptor (GPR41/GPR43) activation, and direct effects on mitochondrial metabolism [173,174]. Critically, SCFAs could upregulate the PINK1/Parkin mitophagy pathway, potentially enhancing clearance of damaged mitochondria and improving mitochondrial quality control [175,176]. Butyrate treatment has been shown to increase PINK1 expression in neuronal cells, promote Parkin recruitment to depolarized mitochondria, and enhance autophagic flux [175,177]. These effects might directly counteract the PINK1 suppression observed in our reconstructed OSA pathway.

Conversely, PINK1 deficiency has been reported to alter gut microbiome composition in Parkinson’s disease models, suggesting a bidirectional relationship between mitochondrial function and microbial community structure [178,179]. PINK1 knockout mice show shifts in gut bacterial populations, reduced SCFA production, increased intestinal permeability, and systemic inflammation [178,180]. These features are remarkably similar to those observed in OSA patients. This suggests a potential vicious cycle: OSA-associated intermittent hypoxia → HIF1A activation → HEY1 induction → PINK1 suppression → altered microbiome composition → reduced SCFA production → further impairment of mitochondrial quality control [134,135,178].

The gut–brain axis represents an increasingly recognized pathway for bidirectional communication between the gastrointestinal system and the central nervous system. This axis is mediated by neural (vagus nerve), endocrine (gut hormones), immune (cytokines), and metabolic (SCFAs, tryptophan metabolites) signals [181,182]. In the context of OSA, this axis may be particularly important. OSA patients show altered gut microbiome composition characterized by reduced microbial diversity, decreased abundance of SCFA-producing bacteria (*Faecalibacterium*, *Roseburia*, *Eubacterium*), and increased pro-inflammatory species [183,184]. These microbial shifts correlate with OSA severity, metabolic complications, and cognitive impairment [183,185]. This suggests that microbiome dysbiosis may contribute to disease pathophysiology rather than merely reflecting secondary consequences of sleep disruption.

Formulation Strategies and Therapeutic Delivery Considerations. Following in vitro validation of PFL-112’s dual-kinase activity and target engagement, future formulation development will need to consider optimal delivery strategies based on experimentally confirmed therapeutic mechanisms. If primary therapeutic benefit derives from central nervous system effects (circadian regulation, neuroprotection), formulation strategies should prioritize blood–brain barrier penetration and neural tissue distribution. PFL-112’s favorable lipophilicity (Log P ~4.0) and oral bioavailability (0.55) support this approach. Conversely, if peripheral mechanisms (gut–immune–metabolic signaling, systemic inflammation modulation) prove therapeutically significant, targeted delivery strategies may warrant investigation. These could include enteric-coated formulations, nanoparticle encapsulation for gut-specific release, or lymphatic targeting.

If PINK1 function is closely tied to gut microbiome health and SCFA signaling, oral administration of PFL-112—rather than systemic or central nervous system delivery—may offer advantages. Gut-targeted delivery could achieve high local concentrations in intestinal tissues where PINK1 expression influences epithelial barrier function, immune regulation, and microbiome composition [178,186]. Modified-release formulations designed for colonic drug delivery could maximize exposure in regions with high SCFA production and dense microbial populations [187,188]. This approach might enhance therapeutic effects while minimizing systemic exposure and off-target effects. PFL-112’s Lipinski compliance, absence of P-glycoprotein efflux, and moderate lipophilicity provide formulation flexibility for multiple delivery approaches.

Moreover, combination approaches pairing PFL-112 with prebiotics (fiber substrates promoting SCFA-producing bacteria), probiotics (beneficial bacterial strains), or postbiotics (purified SCFAs or microbial metabolites) may offer synergistic benefits [189,190]. By simultaneously enhancing PINK1 function through direct kinase modulation (via PFL-112) and through indirect microbiome-mediated pathways (via SCFA upregulation), such combination strategies could potentially achieve more robust and sustained therapeutic effects than either approach alone [175,189].

### 4.9. Neural Tissue Validation and the Immune–Metabolic Dimension: Dual Evidence for Neuroprotective and Immunomodulatory Therapeutic Strategies

Our identification of CK1δ-PINK1 co-expression and functional linkage in Jurkat T cells provides an additional mechanistic connection with direct relevance to OSA pathophysiology. OSA is increasingly recognized as a condition involving immune dysfunction, characterized by chronic low-grade inflammation, altered T-cell populations, increased pro-inflammatory cytokines (TNF-α, IL-6, IL-1β), and impaired immune surveillance [191,192]. T cells from OSA patients show altered phenotypes, including increased activation markers, skewed CD4+/CD8+ ratios, and elevated oxidative stress markers [191,193].

The co-expression of CK1δ and PINK1 in T cells suggests that the circadian–mitochondrial axis identified in our network analysis operates within immune cells. This potentially links circadian disruption to immune dysfunction in OSA. CK1δ regulates circadian rhythms in T cells, influencing the timing of immune responses, cytokine production, and lymphocyte trafficking [194,195]. Circadian misalignment—a cardinal feature of OSA—disrupts these rhythms. This leads to inappropriate timing of immune activation and contributes to the chronic inflammatory state observed in OSA patients [195,196].

Simultaneously, PINK1 maintains mitochondrial quality control in T cells, which are highly metabolically active and dependent on efficient mitochondrial function for proliferation, differentiation, and effector function [197,198]. T-cell activation triggers dramatic increases in energy demand. This requires robust mitochondrial biogenesis and function to support biosynthetic programs [198]. PINK1 deficiency impairs T-cell metabolism, reducing proliferative capacity, altering cytokine production, and increasing susceptibility to apoptosis under metabolic stress [197,199].

The convergence of CK1δ and PINK1 dysfunction in T cells could thus contribute to immune dysregulation observed in OSA through complementary mechanisms. Circadian misalignment disrupts temporal coordination of immune responses. Meanwhile, mitochondrial dysfunction impairs the metabolic capacity required for effective immune function. Dual CK1δ/PINK1 modulation with PFL-112 could potentially address both aspects simultaneously, normalizing circadian timing in immune cells while enhancing mitochondrial health and metabolic resilience [195,197,198].

Neural Tissue Validation Substantiates Neuroprotective Claims. While the Jurkat T-cell co-expression analysis addressed the understudied brain–gut–immune axis in OSA, neuroprotective therapeutic claims require direct neural tissue evidence. To validate the relevance of the CK1δ-PINK1 axis to OSA-associated neuroprotection, we examined tissue-specific co-expression patterns across brain regions implicated in OSA pathology. Analysis using Network Assistant 3 revealed robust CK1δ-PINK1 protein–protein interactions in hippocampus, frontal cortex, hypothalamus, cerebellum, and cerebellar hemisphere (Supplementary Table S1). This demonstrates that the signaling axis is actively expressed in neural tissues vulnerable to intermittent hypoxia-induced damage.

The hippocampus, critical for memory consolidation and spatial learning, exhibits structural atrophy and functional impairment in OSA patients. The frontal cortex, essential for executive function and decision-making, shows documented gray matter volume reductions in severe OSA. The hypothalamus serves as the master circadian pacemaker and is directly disrupted by chronic intermittent hypoxia. The cerebellum and cerebellar hemisphere, important for motor coordination and cognitive processing, display volumetric changes in neuroimaging studies of OSA. Human Protein Atlas data confirm that both CSNK1D (CK1δ) and PINK1 show robust expression across all these brain regions, with CSNK1D displaying ubiquitous high expression and PINK1 showing consistent detection across neural tissues.

This neural tissue-specific validation, combined with extensive functional annotation from UniProt documenting CK1δ’s roles in synaptic transmission, tau phosphorylation, dopaminergic signaling, and neurite outgrowth, provides strong molecular support for the neuroprotective potential of dual CK1δ/PINK1 modulation. The convergence of evidence from both peripheral immune cells (T cells) and central neural tissues demonstrates that the CK1δ-PINK1 signaling axis operates across multiple pathologically relevant cellular contexts in OSA. This supports a multi-dimensional therapeutic strategy addressing both immune–metabolic dysregulation and neurodegeneration.

Connecting Immune and Neural Dimensions Through the Gut–Brain Axis. The T-cell connection also strengthens the microbiome–gut–brain axis hypothesis. Gut-associated lymphoid tissue (GALT) represents the largest immune compartment in the body, housing >70% of all immune cells and serving as a critical interface between the host immune system and gut microbiota [200,201]. T cells in GALT are continuously exposed to microbial signals, including SCFAs, which modulate T-cell differentiation, cytokine production, and regulatory T-cell (Treg) development [201,202].

The finding that CK1δ and PINK1 are functionally linked in T cells, combined with evidence that both kinases interact with microbiome-associated pathways and are robustly expressed in vulnerable brain regions, suggests that therapeutic modulation could achieve multi-level efficacy. Gut-targeted delivery of PFL-112 could modulate T-cell function at the gut–immune interface where microbiome signals are most concentrated [178,200,201,202]. Simultaneously, systemic delivery leveraging PFL-112’s favorable lipophilicity (Log P ~4.0) and oral bioavailability (0.55) could achieve blood–brain barrier penetration for direct neuroprotective effects in hippocampus, cortex, hypothalamus, and cerebellum.

This dual evidence base—immune–metabolic dysregulation in peripheral T cells and neuropathological vulnerability in central neural tissues—provides complementary mechanistic support for PFL-112 as a multi-dimensional therapeutic approach. The compound’s ability to engage both CK1δ and PINK1 across diverse cellular contexts positions it to address the multisystem pathophysiology of OSA, potentially improving outcomes beyond what single-target or single-tissue approaches could achieve.

### 4.10. Therapeutic Implications for OSA Management: CPAP Complementary Therapeutic Potential with or Beyond CPAP

Finally, the potential therapeutic implications for OSA management merit attention, particularly the synergistic opportunities with existing treatment modalities. Current OSA treatment relies predominantly on continuous positive airway pressure (CPAP) devices, which mechanically maintain airway patency during sleep but do not address underlying molecular pathophysiology [7,8]. While CPAP effectively reduces apneic events and improves oxygenation, adherence remains problematic (30–50% non-adherence rates), and many patients continue to experience residual daytime sleepiness, cognitive impairment, and metabolic dysfunction despite adequate mechanical treatment [7,49]. Alternative approaches, including oral appliances and surgical interventions, offer benefits for selected patients but similarly fail to address systemic molecular dysregulation [8].

Mechanistic basis for CPAP–pharmacotherapy synergy. The rationale for combining device-based and pharmacological interventions stems from their complementary mechanisms of action, addressing distinct but interconnected pathological domains. CPAP primarily targets the mechanical/respiratory dimension by maintaining upper airway patency, thereby eliminating obstructive respiratory events and restoring continuous oxygenation during sleep. This intervention immediately addresses the proximate cause of hypoxemia and sleep fragmentation. However, CPAP does not directly modulate the molecular/systemic dimension of OSA pathophysiology—specifically, the circadian dysregulation, mitochondrial dysfunction, chronic inflammation, and metabolic disturbances that accumulate over years of disease and may persist even after mechanical correction of airway obstruction [7,49].

Dual CK1δ/PINK1 modulation, as exemplified by our third-generation compound PFL-112, addresses precisely these residual molecular deficits that CPAP leaves untreated. CK1δ inhibition has been shown to realign circadian timing, modulate sleep architecture, and reduce neuroinflammatory signatures—processes that align closely with the pathological domains highlighted by our comorbidity network [36,40,79]. Small-molecule CK1δ inhibitors can advance circadian phase, enhance slow-wave sleep, and improve sleep continuity in preclinical models, suggesting potential to address the fragmented sleep architecture characteristic of OSA independently of airway mechanics [40,79,80]. These effects could prove particularly valuable for patients with residual excessive sleepiness despite adequate CPAP treatment—a common clinical challenge affecting quality of life and cardiovascular outcomes in an estimated 10–20% of CPAP-adherent patients [7,49].

Addressing the “molecular memory” of chronic OSA. An important consideration supporting combination therapy is that chronic intermittent hypoxia, even when subsequently corrected by CPAP, may establish persistent molecular alterations—a form of “pathological memory”—in circadian clock function, mitochondrial quality control, and inflammatory signaling. Epidemiological studies demonstrate that cardiovascular and neurocognitive risks in OSA patients remain elevated even with years of consistent CPAP use, suggesting that restoration of normal oxygenation alone is insufficient to reverse all accumulated damage [7,49]. Pharmacological intervention targeting CK1δ and PINK1 could theoretically help “reset” these dysregulated molecular programs, potentially accelerating recovery and reducing long-term complications. In this context, PFL-112 would not replace CPAP but rather act as a molecular adjunct to maximize therapeutic benefit by addressing both the cause (airway obstruction via CPAP) and the consequences (molecular dysregulation via dual-kinase modulation).

Temporal and mechanistic complementarity. The synergy between CPAP and dual-kinase inhibition extends to their temporal pharmacodynamics. CPAP provides immediate benefits during sleep (maintained airway, restored oxygenation) but offers no protection during waking hours. Conversely, systemic pharmacotherapy with compounds like PFL-112 would provide continuous 24 h modulation of circadian and mitochondrial pathways, potentially improving daytime function, metabolic regulation, and chronic inflammatory tone that persist beyond the sleeping period. This temporal complementarity suggests that combination therapy could provide more comprehensive coverage of the full circadian cycle, addressing both nocturnal respiratory events and daytime molecular dysregulation.

Furthermore, by reducing chronic HIF1A hyperactivation through CK1δ inhibition, dual-kinase modulators may enhance the efficacy of CPAP itself. Chronic intermittent hypoxia is known to induce maladaptive vascular and neural remodeling that can persist and potentially worsen treatment responsiveness over time. Early pharmacological intervention alongside CPAP initiation might prevent or reverse these structural changes, improving CPAP efficacy and reducing the development of treatment-resistant complications.

Implications for treatment-resistant and residual disease. Dual modulation of CK1δ and PINK1 further extends the therapeutic spectrum toward mitochondrial stabilization and neuroprotection, addressing cognitive decline and metabolic dysfunction commonly observed in OSA patients [18,19,20,37,41]. PINK1 enhancement through reduced HEY1-mediated suppression (via upstream CK1δ inhibition) or through direct positive modulation could improve mitochondrial quality control, reduce oxidative stress, and protect neurons from the cumulative damage imposed by chronic intermittent hypoxia [23,37,131]. This neuroprotective dimension is particularly important given the strong association between OSA and dementia risk, which persists even after controlling for other cardiovascular and metabolic risk factors and despite CPAP treatment [19,20]. For the subset of patients who develop cognitive impairment before OSA diagnosis or who have already accumulated significant neural damage, pharmacological neuroprotection may represent the only viable strategy to slow or reverse decline.

Integrative multi-system intervention. With OSA increasingly recognized as a multisystem disorder rather than an isolated breathing phenomenon—affecting cardiovascular, metabolic, immune, and neurological systems through interconnected molecular mechanisms [3,4,38,39]—pharmacological interventions targeting upstream molecular modules represent a promising complement to device-based treatments such as CPAP. Combination therapy pairing CPAP (to address mechanical airway obstruction and restore oxygenation) with PFL-112 (to address molecular circadian–mitochondrial dysregulation) may offer synergistic benefits, potentially improving treatment outcomes, reducing long-term complications, and enhancing quality of life beyond what either approach achieves alone [7,40,41]. This paradigm shift from purely mechanical intervention to integrated mechanical–pharmacological management mirrors successful approaches in other complex diseases (e.g., heart failure management combining device therapies with neurohormonal modulation).

Future formulation considerations and the gut microbiome hypothesis. The potential for gut-targeted delivery further enhances the therapeutic appeal of this approach, though this remains a hypothesis requiring experimental validation and will be explored comprehensively in a companion manuscript. Oral formulations designed for colonic release could theoretically achieve high local concentrations at the gut–immune–microbiome interface, potentially modulating T-cell function, promoting short-chain fatty acid (SCFA) production, and restoring healthy microbiome composition while minimizing systemic drug exposure and potential off-target effects [178,187,188]. This route of administration offers practical advantages (patient convenience, non-invasive, suitable for chronic administration) while potentially amplifying therapeutic efficacy through microbiome-mediated mechanisms [189,190]. However, whether gut-targeted delivery would prove superior to systemic delivery for OSA management depends on experimental validation of the relative contributions of peripheral (gut–immune) versus central (neural) mechanisms to therapeutic benefit, questions that remain open and warrant systematic investigation.

### 4.11. Limitations and Future Directions

Several important limitations must be acknowledged. First, our findings rest entirely on computational and network-based analyses; experimental validation is essential before drawing firm conclusions about therapeutic potential. In vitro kinase assays measuring IC_50_ values for CK1δ and PINK1 inhibition by ICLID and PFLID represent the immediate next step, along with kinome-wide selectivity profiling to assess off-target effects [158,161]. Cellular assays in models relevant to OSA pathophysiology—including intermittent hypoxia exposure, circadian rhythm analysis, mitophagy assessment, and oxidative stress quantification—will be required to validate predicted functional effects [37,203,204].

Second, the translation from computational binding scores (kcal/mol) to biochemical activity (IC_50_, K_d_) and ultimately to cellular and organismal effects involves substantial uncertainty. Docking scores provide valuable rank-ordering of compounds but cannot reliably predict absolute binding affinities or account for factors such as protein flexibility, solvent effects, entropy, and induced-fit binding [27,35,88]. Only experimental validation can definitively establish whether the predicted binding advantages of ICLID and PFLID translate into superior functional activity.

Third, drug-like properties beyond target binding—including solubility, membrane permeability, metabolic stability, plasma protein binding, and toxicity—will determine whether these compounds can advance to preclinical development [102,205]. The hybrid structures of ICLID and PFLID, while designed to optimize binding, may present challenges for pharmaceutical formulation or show unfavorable pharmacokinetic profiles. Medicinal chemistry optimization guided by structure–activity relationships will likely be necessary to achieve compounds suitable for in vivo evaluation [205].

Fourth, in vivo validation in animal models of OSA will be essential but challenging. Chronic intermittent hypoxia (CIH) models in rodents recapitulate key features of OSA, including sleep fragmentation, oxidative stress, metabolic dysfunction, and cognitive impairment [203,204]. However, these models do not fully capture the mechanical airway obstruction and complex pathophysiology of human OSA [206]. Nevertheless, CIH models provide valuable platforms for assessing whether ICLID/PFLID can improve circadian rhythm parameters, reduce neuroinflammation, preserve mitochondrial function, and prevent cognitive decline—outcomes directly relevant to the therapeutic hypothesis [203,204].

Finally, the broader applicability of our five-tier comorbidity-driven workflow deserves exploration. While developed for OSA, this approach may prove valuable for other complex multimorbid conditions where molecular signatures remain poorly defined, and bottom-up omics approaches have struggled to identify coherent therapeutic targets. Chronic obstructive pulmonary disease (COPD), heart failure, metabolic syndrome, and chronic kidney disease all share features that could benefit from comorbidity-anchored target identification: extensive multimorbidity, heterogeneous molecular profiles, and limited success with single-target therapies [5,13,14]. Applying our workflow to these conditions could reveal additional disease modules and therapeutic opportunities.

## 5. Conclusions

This study establishes a top-down, comorbidity-driven framework for drug discovery in complex diseases like Obstructive Sleep Apnea (OSA). By pivoting from broad omics data to clinically validated comorbidity networks, we identified a novel disease module centered on CK1δ and PINK1. This molecular axis mechanistically bridges circadian rhythm disruption and neurodegenerative processes through a CK1δ-HIF1A-HEY1-PINK1 signaling cascade.

Our therapeutic approach utilizes a dual-kinase “pincer strategy.” By simultaneously modulating circadian regulation (via CK1δ) and mitochondrial quality control (via PINK1), we provide a coordinated attack on the underlying pathophysiology of OSA rather than merely treating its symptoms. While natural alkaloids from *Nigella sativa* and the inhibitors (PF-670462 and IC261, referred to here as first generation) provided the initial structural template, they lacked the potency required for clinical translation.

To address these limitations, we developed third-generation, ADME-proofed inhibitors, specifically ICL-89 and PFL-112. These compounds represent a significant advancement over second-generation leads (ICLID/PFLID). ICL-89 and PFL-112 maintain exceptional dual-target binding affinities while achieving the metabolic stability and oral bioavailability necessary for in vivo efficacy. These candidates successfully overcome the common “bottom-up” hurdle of translating computational hits into viable drug leads.

In conclusion, we identified the CK1δ-PINK1 axis as a mechanistic bridge between circadian disruption and neurodegeneration, characterized natural dual kinase modulators, and designed next-generation inhibitors with enhanced predicted efficacy. Ultimately, this five-tier modeling workflow—from comorbidity network analysis to ADME optimization—offers a generalizable template for precision medicine in multimorbid conditions. The progression of ICL-89 and PFL-112 into preclinical models will be the immediate priority to validate this “pincer strategy” in restoring circadian rhythm and neuronal protection.

## Figures and Tables

**Table 1:** Molecular docking scores of natural alkaloids, reference inhibitors, and multi-generational rationally designed dual inhibitors against CK1δ and PINK1. Docking calculations were performed using AutoDock Vina with crystal structures of CK1δ (PDB: 3UYS) and PINK1 (PDB: 5OAT). Binding affinities are reported as Vina scores (kcal/mol), where more negative values indicate stronger predicted binding. The binding site volume (Å^3^) represents the search space used for docking calculations. Natural compounds from *Nigella sativa* comprise the 0th generation scaffolds, including Nigeglanine (GLA), Nigellicine (LIC), and Nigellidine (LID), with Melatonin (MLT) as a circadian reference compound. First-generation reference inhibitors IC261 and PF-670462 provided synthetic pharmacophoric templates for rational drug design. Second-generation dual inhibitors ICLID and PFLID (shown in red and bold) represent initial hybrid designs combining structural features from both natural and synthetic sources, demonstrating enhanced binding affinities compared to parent compounds but exhibiting significant ADME liabilities. Third-generation ADME-optimized inhibitors ICL and PFL (shown in blue and bold) were developed through systematic structure-guided modifications to address pharmacokinetic deficiencies identified in ICLID and PFLID, followed by re-docking validation. Notably, PFL achieved the strongest predicted binding affinities across both targets (CK1δ: −10.8 kcal/mol; PINK1: −11.2 kcal/mol) while maintaining superior drug-likeness properties, demonstrating that ADME optimization can preserve or even enhance target engagement. The progressive improvement in binding affinity from reference inhibitors through second-generation hybrids to third-generation ADME-proofed compounds validates the multi-stage rational design and pharmaceutical optimization strategy employed in this study.

Name: PDB (Kinase Name)	Vina Score (kcal/mol)	Volume (A^3^)
GLA:3UYS (CK1δ)	−6.7	877
LIC:3UYS (CK1δ)	−7.1	637
LID:3UYS (CK1δ)	−8.0	637
MLT:3UYS (CK1δ)	−6.8	877
GLA:5OAT (PINK1)	−7.1	2559
LIC: 5OAT (PINK1)	−8.6	2559
LID: 5OAT (PINK1)	−7.8	2559
MLT: 5OAT (PINK1)	−6.9	2559
**ICLID**:3UYS (CK1δ):	**−8.5**	637
IC261:3UYS (CK1δ)	−6.5	637
**ICLID**: 5OAT (PINK1):	**−10.3**	3727
IC261: 5OAT (PINK1)	−7.3	2559
**PFLID**:3UYS (CK1δ):	**−9.8**	637
PF670462:3UYS (CK1δ)	−7.9	637
**PFLID**: 5OAT (PINK1):	**−10.0**	3727
PF670462: 5OAT (PINK1)	−7.8	2559
**ICL**:3UYS (CK1δ):	**−7.2**	637
**ICL**: 5OAT (PINK1):	**−8.9**	3727
**PFL**:3UYS (CK1δ):	**−10.8**	651
**PFL**: 5OAT (PINK1):	**−11.2**	3727

Names and the values of the rationally designed compounds are in bold. In red are second generation, in blue are third generation.

## Data Availability

The data presented in this study are available on request from the corresponding author. Restrictions apply to the availability of computational models and intermediate datasets due to ongoing research and intellectual property considerations.

## Supplementary Materials

The following supporting information can be downloaded at https://www.mdpi.com/article/10.3390/biomedicines14010181/s1. Table S1: Neural tissue expression of CK1δ (CSNK1D) and PINK1. File S1: Extended Methodological Details, Workflows, and Rationales.

## Abbreviations

The following abbreviations are used in this manuscript:

Disease	Clinical Terms
OSA	Sleep Apnea, Obstructive (MeSH)
COPD	Pulmonary Disease, Chronic Obstructive (MeSH)
CoMs	Comorbidity (MeSH)
CPAP	Continuous Positive Airway Pressure (MeSH)
Molecular Biology/Genetics	
CK1δ/CSNK1D	Casein Kinase 1 (MeSH) (gene symbol CSNK1D retained in text, but MeSH term used in keywords)
HIF1A	Hypoxia-Inducible Factor 1, alpha Subunit (MeSH)
PINK1	PTEN-Induced Kinase 1 (MeSH)
HEY1	Hairy and Enhancer-of-Split-Related Protein 1 (MeSH)
Bioinformatics	Omics
GO	Gene Ontology (MeSH Supplementary Concept)
KEGG	Biological Pathways (MeSH)
GWAS	Genome-Wide Association Study (MeSH)
PPI	Protein Interaction Maps (MeSH)
FDR	False Discovery Rate (MeSH)
GEO	Gene Expression Profiling (MeSH)
Databases	Structural Biology
PDB/RCSB	Protein Data Bank (MeSH Supplementary Concept)
UniProtKB	Protein Sequence Databases (MeSH)
CTD	Toxicogenomics (MeSH)
Chemical Compounds	Drugs
Melatonin (MLT)	Melatonin (MeSH)
Nigellidine (LID)	Nigellidine (Supplementary Concept)
Nigellicine (LIC)	Nigellicine (Supplementary Concept)
Nigeglanine (GLA)	Plant Extracts/*Nigella sativa* (MeSH)
CID	Chemical Identifiers (MeSH)
